# Generation and Characterization of a Bivalent HIV-1 Subtype C gp120 Protein Boost for Proof-of-Concept HIV Vaccine Efficacy Trials in Southern Africa

**DOI:** 10.1371/journal.pone.0157391

**Published:** 2016-07-21

**Authors:** Carlo Zambonelli, Antu K. Dey, Susan Hilt, Samuel Stephenson, Eden P. Go, Daniel F. Clark, Mark Wininger, Celia Labranche, David Montefiori, Hua-Xin Liao, Ronald I. Swanstrom, Heather Desaire, Barton F. Haynes, Andrea Carfi, Susan W. Barnett

**Affiliations:** 1 GSK Vaccines (formerly Novartis Vaccines), 45 Sidney Street, Cambridge, MA, 02139, United States of America; 2 Department of Chemistry, University of Kansas, Lawrence, KS, 66047, United States of America; 3 Department of Surgery, Duke University Medical Center, Durham, North Carolina, United States of America; 4 Duke Human Vaccine Institute, Duke University Medical Center, Durham, North Carolina, United States of America; 5 University of North Carolina, Chapel Hill, NC, United States of America; Emory University, UNITED STATES

## Abstract

The viral envelope glycoprotein (Env) is the major target for antibody (Ab)-mediated vaccine development against the Human Immunodeficiency Virus type 1 (HIV-1). Although several recombinant Env antigens have been evaluated in clinical trials, only the surface glycoprotein, gp120, (from HIV-1 subtype B, MN, and subtype CRF_01AE, A244) used in the ALVAC prime-AIDSVAX gp120 boost RV144 Phase III HIV vaccine trial was shown to contribute to protective efficacy, although modest and short-lived. Hence, for clinical trials in southern Africa, a bivalent protein boost of HIV-1 subtype C gp120 antigens composed of two complementary gp120s, from the TV1.C (chronic) and 1086.C (transmitted founder) HIV-1 strains, was selected. Stable Chinese Hamster Cell (CHO) cell lines expressing these gp120s were generated, scalable purification methods were developed, and a detailed analytical analysis of the purified proteins was conducted that showed differences and complementarity in the antigenicity, glycan occupancy, and glycan content of the two gp120 molecules. Moreover, mass spectrometry revealed some disulfide heterogeneity in the expressed proteins, particularly in V1V2-C1 region and most prominently in the TV1 gp120 dimers. These dimers not only lacked binding to certain key CD4 binding site (CD4bs) and V1V2 epitope-directed ligands but also elicited reduced Ab responses directed to those epitopes, in contrast to monomeric gp120, following immunization of rabbits. Both monomeric and dimeric gp120s elicited similarly high titer Tier 1 neutralizing Abs as measured in standard virus neutralization assays. These results provide support for clinical evaluations of bivalent preparations of purified monomeric TV1.C and 1086.C gp120 proteins.

## Introduction

HIV-1 infection and acquired immunodeficiency syndrome (AIDS) represent a major public health concern. HIV/AIDS is most prevalent in sub-Saharan Africa, where almost 70% of all HIV-infected people live. HIV-1 subtype C accounts for over 95% of infections in southern African [1] and over 50% of HIV-1 infections globally [2]. While recent successes in controlling infection and disease have been achieved by increased access to antiretroviral treatment (ART), there are yet millions of people who do not receive treatment [3]. Hence, the development of an efficacious vaccine targeting HIV-1 subtype C endemic in this region would have a significant social and economic impact [4].

Diverse HIV vaccines have been tested in early phase clinical trials [5]. The earliest of these trials focused on recombinant gp120 antigens for the elicitation of antibody (Ab) responses [6–11]. While safe and immunogenic, these gp120 vaccines failed to show protection in two pivotal Phase 3 HIV vaccine trials [12, 13]. Subsequent approaches adopted vaccines designed to preferentially stimulate cytolytic CD8+ T cell (CTL) immunity. These trials also failed to show protection, and a potential enhancement of disease was reported in some individuals [14, 15]. Moreover, the more recent HVTN505 trial using a multivalent recombinant DNA prime and adenovirus boost vaccine approach failed to protect against HIV [16].

The first evidence of HIV vaccine efficacy came from the RV144 Phase 3 trial in Thailand [17]. This trial tested a recombinant canarypox prime followed by a bivalent gp120 boost. The trial showed modest efficacy (31%, 95% CI 1.1 to 52.1, P = 0.04) based on analysis of the clinically relevant modified intent to treat (mITT) population. Notably, the level of protection over the first year was 60% coinciding with peak vaccine immunogenicity. Protection waned over time, in parallel with decreasing levels of the vaccine-induced immune responses [18]. Subsequent correlates of transmission risk analysis showed that Abs directed against the V1V2 region of the Env were associated with reduced risk of infection in vaccinees [19], and molecular sieve analysis showed that specific epitopes in V2 were subjected to immune pressure by the vaccine [20]. Analyses of the quality and functionality of Abs demonstrated that anti- V1V2 Abs of the IgG3 subclass were associated with protection [21] showing increased poly-functionality [22].

The Pox-Protein Public Private Partnership or “P5” was formed in 2010 to follow-up on the clinical results of RV144 [23]. The P5 proposed to evaluate a vaccine similar to the one used in RV144, but adapted to target the most common HIV subtype in South Africa (subtype C). The prime/boost vaccine regimen under consideration is the ALVAC-HIV (vCP2438) prime and bivalent subtype C gp120/MF59 boost, composed of two subtype C HIV-1 Env proteins, using a potent adjuvant and an additional booster dose beyond that administered in RV144.

The TV1.C and 1086.C gp120 antigens were selected in consultation with a group of HIV vaccine experts to provide a bivalent subtype C protein boost component. Here we report the generation of stable CHO cell lines expressing these two gp120s, development of a scalable purification process, a comprehensive analytical characterization of the purified gp120s, and confirmation of the immunogenicity of the candidate proteins. These studies serve as a foundation for cGMP manufacture of these candidates for post-RV144 clinical evaluations.

## Materials & Methods

Reagents are listed in S1 File.

### Generation of CHO stable cell lines

Methods used to generate and evaluate stable cell lines expressing gp120 are provided in S1 File) and summarized in S1 Fig.

### Purification of gp120 proteins

Initially, gp120 proteins were purified using a three-step purification process involving Galanthus Nivalis-Agarose (GNA) affinity column, anion-exchange DEAE column and a final ceramic hydroxyapatite (CHAP) column, as described previously [24]. Subsequently, gp120 proteins expressed via stable CHO cell lines were purified by a two-step ion-exchange purification processes involving Fractogel SO_3_—column (cation exchange) and DEAE (anion exchange) column. Details are described in S1 File.

The gp120 mutants (TV1.C gp120ΔV3, TV1.C gp120ΔV1V2, TV1.C gp120 D368R and 1086.C gp120ΔV3, 1086.C gp120ΔV1V2, 1086.C gp120 D368R) used in serum-mapping studies were generated as described previously [25].

### Antigenicity measurements

Binding affinities of TV1.C and 1086.C gp120 proteins to a gp120 ligands were determined using Surface Plasmon Resonance (SPR, BIAcore 3000). Approximately 200 RU of sCD4 or mAbs were immobilized directly onto a CM5 sensor chip via amine coupling. Varying concentrations of gp120, either TV1.C or 1086.C, were then injected at 100 μl/min and regenerated using either two 60s-injections of Glycine-HCl pH 3.0 or 10mM NaOH pH 10.0. The binding studies were performed at 25°C with PBS (+0.1% BSA) as running buffer. The experimental curves were fitted to a 1:1 Langmuir binding model using BIAevaluation software 3.2 (BIAcore Inc). Binding of TV1.C or 1086.C gp120s to 34 HIV+ sera from South African volunteers was performed using a D7324-capture ELISA, as reported previously [26].

### Glycopeptide and disulfide bond analyses using mass spectrometry

Glycopeptide analyses of TV1.C or 1086.C gp120 monomer samples (~75 μg protein) were performed on partially deglycosylated and non-deglycosylated samples. Samples were partially deglycosylated with endoglycosidase H (EndoH). For partial deglycosylation, samples were denatured with 2 M urea followed by the addition of 2 μL of Endo H (≥5 units/mL) and incubated for 48 hrs at 37°C. Partially deglycosylated and non-deglycosylated samples were denatured with 6 M urea in 50 mM Tris buffer (pH 8.0) containing 3 mM EDTA and were fully reduced using 5 mM TCEP at room temperature (RT) for 1 hr. Following reduction, samples were alkylated with 20 mM IAM at RT for 1 hr in the dark. Excess IAM was quenched with DTT to a final concentration of 25 mM for 20 min at RT. The reduced and alkylated samples were buffer exchanged and concentrated using a 30 kDa MWCO filter (Millipore, Billerica MA) prior to trypsin digestion. Samples were subsequently digested with trypsin (50:1 protein: enzyme ratio) at 37°C and incubated overnight, followed by second trypsin addition under the same conditions. Resulting gp120 digests were subjected to LC/MS/MS analysis using high and low resolution mass spectrometers described in the next paragraph. Glycopeptide identification and analysis of glycosylation site occupancy were performed as described previously [27].

Disulfide bond patterns of the TV1.C and 1086.C gp120 were determined by mapping the disulfide linked peptides with mass spectrometry. Samples containing 75 μg of gp120 protein were alkylated with 10-fold molar excess of 4-vinylpyridine in the dark for an hour at room temperature to cap free cysteines. Alkylated samples were then deglycosylated with 1 μL PNGase F solution (500,000 units/mL) at 37°C for a week. Fully deglycosylated alkylated samples were digested with trypsin (protein to enzyme ratio of 30) overnight. To ensure reproducibility, deglycosylation and subsequent trypsin digestion were performed at least two times. Following tryptic digestion, samples were analyzed using a hybrid linear ion trap Fourier Transform-Ion Cyclotron Resonance (LTQ-FTICR, ThermoScientific, San Jose CA) mass spectrometer coupled with a nanoACQUITY Ultra Performance Liquid Chromatography (UPLC) system (Waters, Milford MA) for high resolution LC/MS/MS analysis and an LTQ Velos mass spectrometer (ThermoScientific, San Jose CA) equipped with electron transfer dissociation (ETD) and a ACQUITY UPLC system for low resolution LC/MS/MS analysis. Chromatographic separation for both low and high resolution LC/MS/MS analyses was performed using mobile phases consisted of solvent A: 99.9% deionized H_2_O + 0.1% formic acid and solvent B: 99.9% CH_3_CN + 0.1% formic acid and a C18 PepMap 300^™^ column (150mm×300 μm i.d. 5 μM, 300Å, ThermoScientific Dionex Sunnyvale, CA). Approximately 5 μl of sample was injected into the column at a flow rate of 5 μl/min using the following gradient: a linear increase to 40% B in 50 minutes then to 90% B in 10 min. The column was held at 90% B for 10 min before re-equilibration to starting conditions. Data were collected in a data dependent acquisition mode in which the five most intense ions in a high resolution survey scan in the FTICR cell were sequentially and dynamically selected for subsequent collision-induced dissociation (CID) in the LTQ linear ion trap. The LTQ Velos mass spectrometer was set up to perform experiments by alternating CID and ETD acquisition. Data dependent acquisition (DDA) was set up to acquire 10 scan events: for every one full MS scan in the mass range, 300–2000 *m/z*, each selected *m/z* in the MS scan were subjected to three MS/MS events- (a) CID, (b) ETD, and (c) CID of the charge reduced precursor in the previous ETD event. The mass spectrometric parameters used for the experiment were: spray voltage 3.0 kV, S-lens value between 45–55%, capillary temperature of 250°C, normalized collision energy of 35% for CID, and the ion-ion reaction for ETD between the precursor ion and the radical anion, fluoranthane, was set at AGC target value of 2x10^5^ and 100 msec ion-ion reaction time. To improve ETD efficiency, supplemental activation was turned on.

### Immunogenicity studies in rabbits

Immunization studies were conducted at Josman LLC (Napa, CA), a USDA licensed research facility (No. 93-R-0260) with a Public Health Service (PHS) Assurance from the NIH (No. A3404-01). Five young adult female New Zealand White rabbits were used in each study group. Rabbits were immunized with gp120 proteins using MF59 plus carbopol as previously described [25]. Three immunizations were administered intramuscularly, in the gluteus muscle at weeks 0, 4, and 12. The total protein dosage at each immunization was 25 μg. Serum samples from blood collected prior to the first immunization (pre-bleed) and following each immunization were analyzed for binding and neutralizing Ab responses. The study was fully approved by the Institutional Animal Care and Use Committee at Novartis (approval no. 09 NVD 044.3.3.09) in accordance with the requirements for the humane care and use of animals as set forth in the Animal Welfare Act, the ILAR Guide for the care and Use of Laboratory Animals, and all applicable local, state and federal laws and regulations.

### Env-specific antibody ELISA and avidity measurements

Env-specific binding Ab titers were measured by a standard ELISA assay, and Ab avidity indices were measured using an ammonium thiocyanate (NH_4_SCN) displacement ELISA. Both assays employed the matched gp120 Ags and were performed as previously described [24].

### Virus neutralization assays

Virus neutralization was measured using a well-standardized assay employing HIV pseudoviruses and a luciferase reporter gene assay in TZM-bl cells (Dr. John C. Kappes, Dr. Xiaoyun Wu and Tranzyme, Inc. (Durham, NC)) as reported previously [28–30] and summarized in the S1 File.

### Mapping of epitope-specific antibody binding

To determine epitope-specific Abs in 2wp3 sera from rabbits immunized with gp120 proteins, WT or mutant gp120s were captured onto the ELISA plates using D7324 as previously described [26]. The bound serum Abs were then washed and detected using a goat anti-rabbit IgG Fc antibody conjugated to horseradish peroxidase, and the optical density determined as absorbance at 450 nm using a microplate reader (Molecular Devices).

Percent epitope-directed binding was calculated as the (EC50 of binding to the mutant version of gp120 divided by EC50 of binding to the WT gp120) x 100.

### Statistical analyses

Comparisons among groups were carried out using analysis of variance (1 way ANOVA). A Kruskal-Wallis test was used to analyze differences between multiple epitope-directed groups. For all comparisons, a two-sided p<0.05 was considered statistically significant.

### Nomenclature

The electronic version of this article in Portable Document Format (PDF) in a work with an ISSN or ISBN will represent a published work according to the International Code of Nomenclature for algae, fungi, and plants, and hence the new names contained in the electronic publication of a PLOS article are effectively published under that Code from the electronic edition alone, so there is no longer any need to provide printed copies.

In addition, new names contained in this work have been submitted to IPNI, from where they will be made available to the Global Names Index. The IPNI LSIDs can be resolved and the associated information viewed through any standard web browser by appending the LSID contained in this publication to the prefix http://ipni.org/. The online version of this work is archived and available from the following digital repositories: PubMed Central and LOCKSS.

## Results

### Selection of HIV-1 subtype C gp120 candidates for post-RV144 clinical trials

In consultation with experts in the HIV vaccine field and based on information available at the time, a list of criteria were established to guide the selection of two gp120 proteins as a boost for future clinical trials for the southern African region (S1 Table). Two gp120s were chosen, one from the South African HIV-1 TV1.C strain isolated during the early chronic phase of infection [31, 32] and another from the HIV-1 1086.C transmitted founder strain from Malawi [33]. The selected gp120s appeared to be complementary being genetically distinct (77.8% amino acid sequence identity) and from HIV-1 strains representing different stages of infection and geographic regions.

### Generation of stable CHO cell lines and scalable purification methods

Stable CHO cell lines expressing gp120s from TV1.C and 1086.C were generated after screening a large numbers of clones (S1 Fig). The presence of a dimeric gp120 fraction was detected by Western blot analysis of the culture supernatants and clones with the highest percentage of monomeric gp120 were selected for further evaluations. TV1.C gp120 CHO clones were selected that expressed greater than 100 mg/l whereas the 1086.C clones expressed at a lower level (20–25 mg/l). A 3–4 fold increase in protein expression was observed at 32°C for 1086.C gp120, (i.e. 60–80 mg/l) (S2 Fig). The top clones were then evaluated over a period of 78 days in continuous re-feed batch cultures with and without G418 selection. Top *clones from both TV1.C and 1086.C were shown to stably express the corresponding gp120s during this time (S3 Fig).

**Fig 1:**
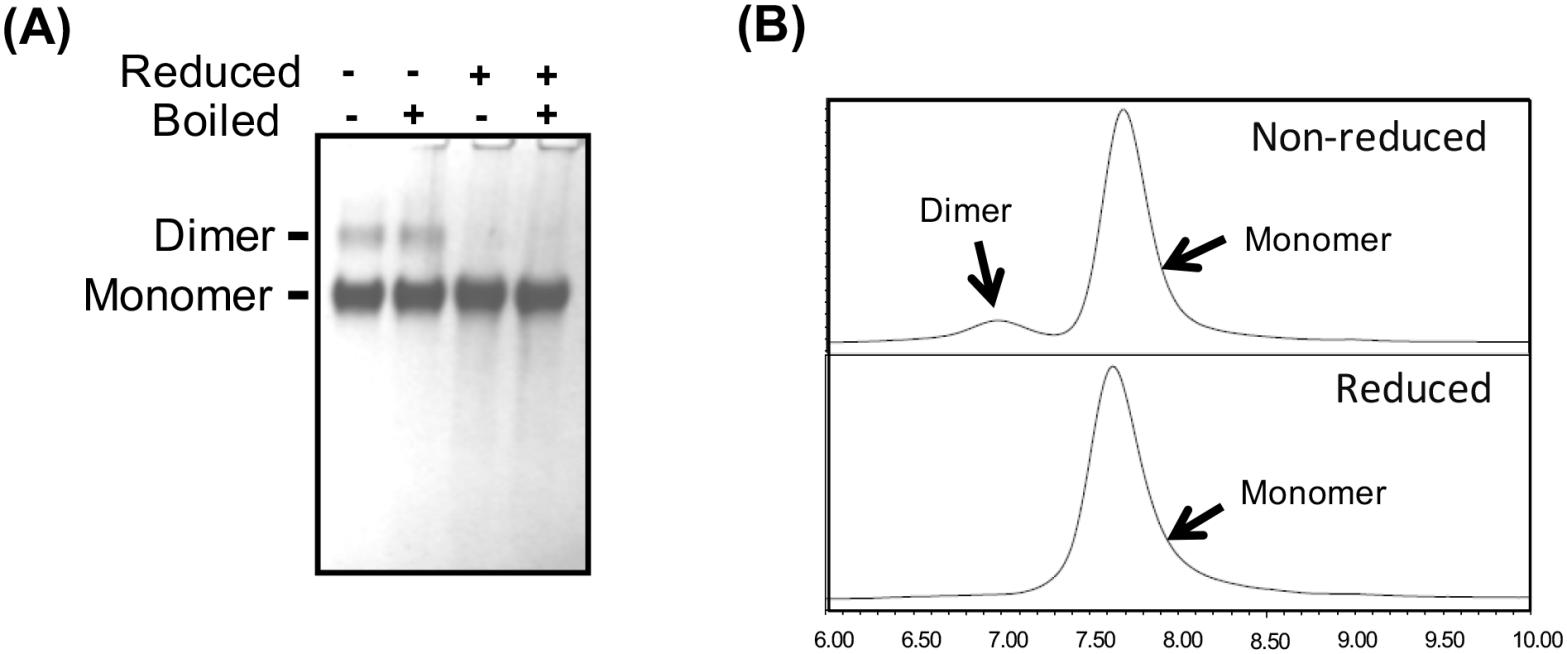
Analysis of purified TV1.C gp120 revealed the presence of a dimeric fraction. Panel A: SDS-PAGE conducted under reduced and non-reduced conditions. Panel B: SEC-HPLC showing non-reduced (upper panel) and reduced (lower panel) conditions. Results confirm the presence of the TV1.C gp120 dimeric form; SEC-HPLC, under reducing, but otherwise native conditions, showed that the reduced gp120 monomer and dimer form a homogenous population. Due to the very low amount of dimeric gp120 protein for 1086.C, comparison of reduced and non-reduced conditions is not shown.

During the initial screening phase, gp120 proteins were purified using GNA (Galanthus nivalis) affinity chromatography followed by polishing with DEAE and CHAP columns [24]. SDS-PAGE analysis showed that monomers and SDS resistant dimers were present in TV1.C preparations (Fig 1) whereas 1086.C gp120 preparations had very low levels of dimers (data not shown). Hence, the addition of a size-exclusion chromatography (SEC) purification step was required for further enrichment of monomers. However, considering that GNA lectin and SEC would not be the preferred methods for future cGMP manufacturing, more suitable purification protocols for these gp120s had to be developed using capture onto a Fractogel EMD SO_3_ resin followed by differential elution of gp120 monomers and dimers using a pH gradient wash. Further polishing on DEAE anion exchange column in flow through mode resulted in highly pure monomeric gp120 protein (>95% purity and >90% monomer content). The 2-step anion exchange purification method was used for the production of proteins analyzed as described in the following sections.

### Glycosylation analysis of the CHO produced 1086.C and TV1.C gp120s

The glycosylation site occupancy and composition of the CHO-expressed TV1.C and 1086.C gp120 monomers was assessed by mass spectrometry (MS) analysis of partially de-glycosylated and non-deglycosylated samples [27]. The tryptic peptides, the potential glycosylation sites and their occupancies are listed in S2 and S3 Tables. For TV1.C gp120, 27 of the 30 potential N-linked glycosylation (PNG) sites were partially occupied whereas the remaining 3 sites (N88, N230 and N339) were fully occupied. The conserved potential O-linked glycosylation site at T499 was also partially occupied (S2 Table). For the 1086.C gp120, 20 PNG sites and the O-linked glycosylation site at T499 had variable occupancy and 3 PNG sites (N88, N187, and N230) were fully occupied (S3 Table).

**Fig 2:**
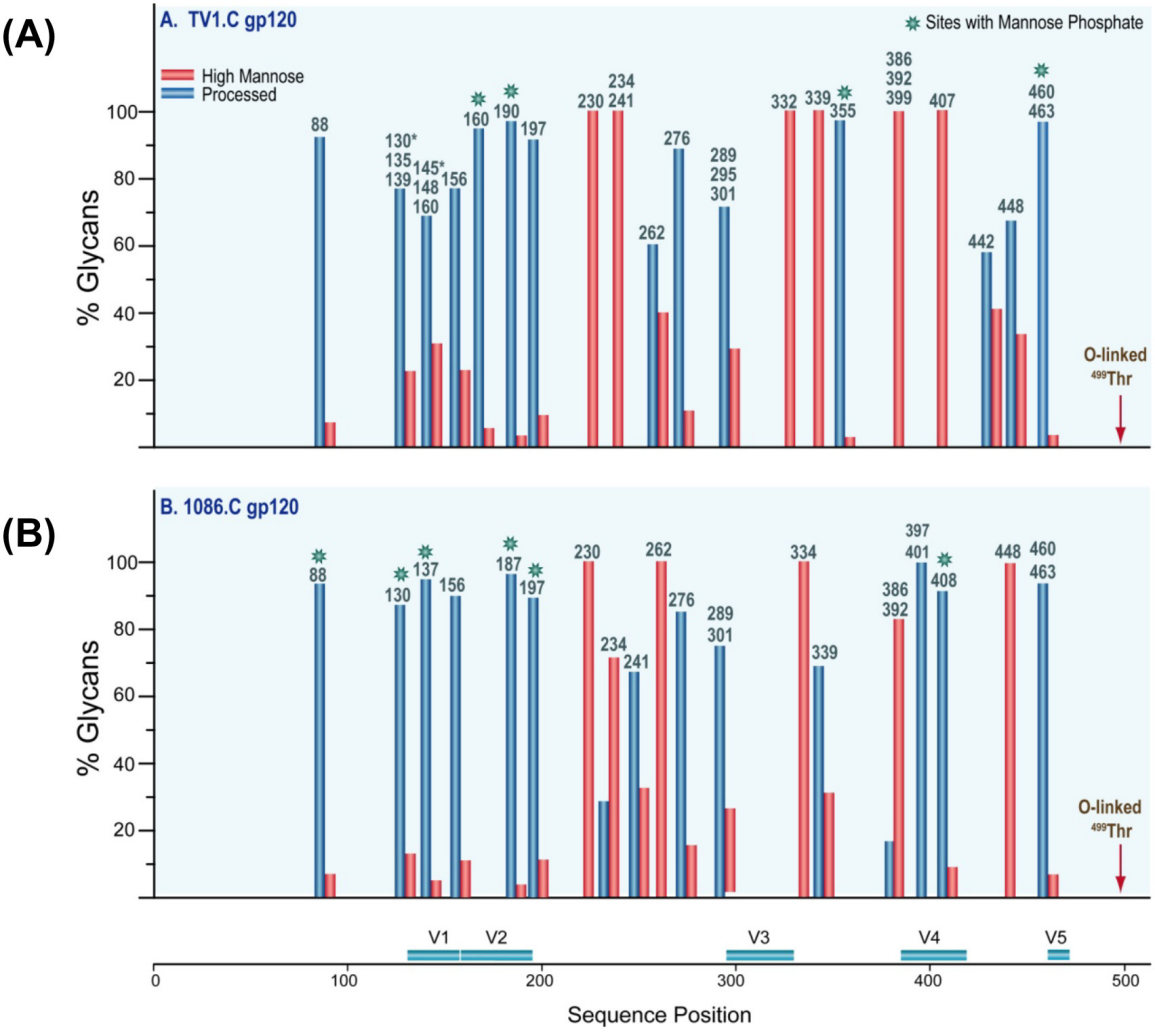
Characterization of potential N-linked glycosylation sites (PNGS). The glycan compositions (in percentages) of TV1.C (A) and 1086.C (B) glycopeptides were sorted and broadly grouped based on criteria described previously [34]. Here, the glycan profile of each glycopeptide with either single or multiple glycosylation sites were represented by a pair of bars denoting the percentage of the type of glycan: high mannose (red) or processed (blue), according to Env sequence position, as indicated in S2 Table (TV1.C gp120) and 3 (1086.C gp120). (See also S2 and S3 Tables for more detailed information on the absolute occupancies at each site). The asterisks denote sites containing mannose phosphate.

For TV1.C gp120, 9 of 30 of the occupied PNG sites were composed of exclusively high-mannose glycans, while the remaining sites contained predominantly processed glycans (Fig 2A). The 1086.C gp120 glycan profile showed that 7 sites had exclusively or primarily high-mannose glycans, and 16 sites had processed glycans (Fig 2B). When the glycan profiles of the CHO-expressed 1086.C gp120 were compared to a prior analysis of 293HEK-expressed 1086.C gp140 [27], some host cell-specific glycosylation differences were noted at sites 234, 241, 276, 289 and 339. These sites contained more processed glycans in the CHO 1086.C gp120 than in the 293HEK- 1086.C gp140s [27]. In contrast, the C1 and V1V2 regions showed almost exclusively processed glycans in both CHO- and 293HEK-expressed gp120s. Other regions were also similar showing high mannose glycans in both preparations.

### Analysis of disulfide linkages

**Fig 3:**
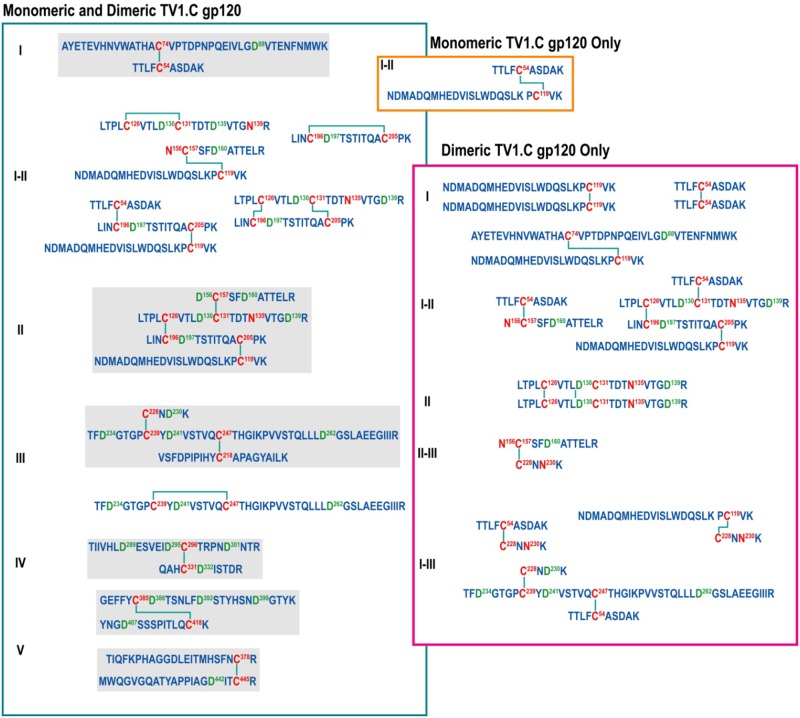
Disulfide bonds identified for TV1.C gp120 monomer and dimer highlighting that the disulfide bonding pattern leading to dimer formation is more complex than what is observed for the monomeric protein. A similar analysis was not performed for 1086.C gp120 as it was only a very minor species in the preparations.

MS was used to investigate the disulfide-bond patterns in fractionated gp120 monomers and dimers. The LC-MS data confirmed the canonical disulfide bond between the conserved 18 cysteines in both TV1.C gp120 and 1086.C gp120 monomers (S4 and S5 Tables). In addition, several non-canonical disulfide bonded peptides were also detected; these are commonly observed in recombinant gp120 and gp140 [35–38] (S6 and S7 Tables). As expected, analysis of the TV1.C gp120 dimeric population showed several additional scrambled inter-molecular disulfide bonds, particularly in the C1-V1V2-C2 region (Fig 3). These results confirmed that gp120 dimer formation occurs via aberrant inter-molecular disulfide bonds as previously described [39].

### Evaluation of the antigenicity of TV1.C and 1086.C gp120s using a panel of mAbs and HIV-1 positive sera from southern Africa

The antigenic properties of the TV1.C and 1086.C gp120 monomers and dimers were evaluated using surface plasmon resonance (SPR). gp120 monomers from both strains bound the soluble CD4 receptor ligand, CD4-Fc (sCD4), and the anti-V3 mouse mAbs, 1B7A6 and 10B6A8, with affinities in the nanomolar range (Table 1). Otherwise, the two gp120s showed different and complementary binding profiles. TV1.C gp120 bound to the 2G12 and PG09 mAbs, while 1086.C gp120 bound to b12, VRC01, CH58, and CH59; neither bound to PG16 and CH01, mAbs, with PG09, specifically recognizing quaternary structures Additionally, the TV1.C gp120 showed increased binding to the 1B7A6 and 10B6A8 mAbs and 1086.C gp120 showed higher affinity binding to sCD4 as compared to TV1.C gp120. In contrast, neither of TV1.C nor 1086.C gp120 dimers exhibited binding to sCD4, even when proteins were injected at the highest concentration (100 nM). Finally, whereas the 1086.C gp120 monomer bound to the anti-V1V2-specific antibodies CH58 and CH59, the dimeric forms of gp120 did not.

**Table 1:** Binding affinities for TV1.C and 1086.C gp120 using various gp120 ligands. Measurements were performed using SPR as described in Methods. For each ligand the target epitope is indicated in brackets.

mAb (epitope)	K_D_ (nM)	
	TV1.C gp120	1086.C gp120
CD4-Fc	148	26.6
b12 (CD4BS)	DNB	88.1
VRC01 (CD4BS)	DNB	93.5
2G12 (Mannose)	75	DNB
1B7A6 (V3)	3.5	30.5
10B6A8 (V3)	3.06	37.5
PG09 (V2-V3)	23.8	DNB
PG16 (V2-V3)	DNB	DNB
CH01 (V2-V3)	DNB	DNB
CH58 (V1V2)	DNB	22.8
CH59 (V1V2)	DNB	33.1

*DNB* = Did not bind

**Fig 4:**
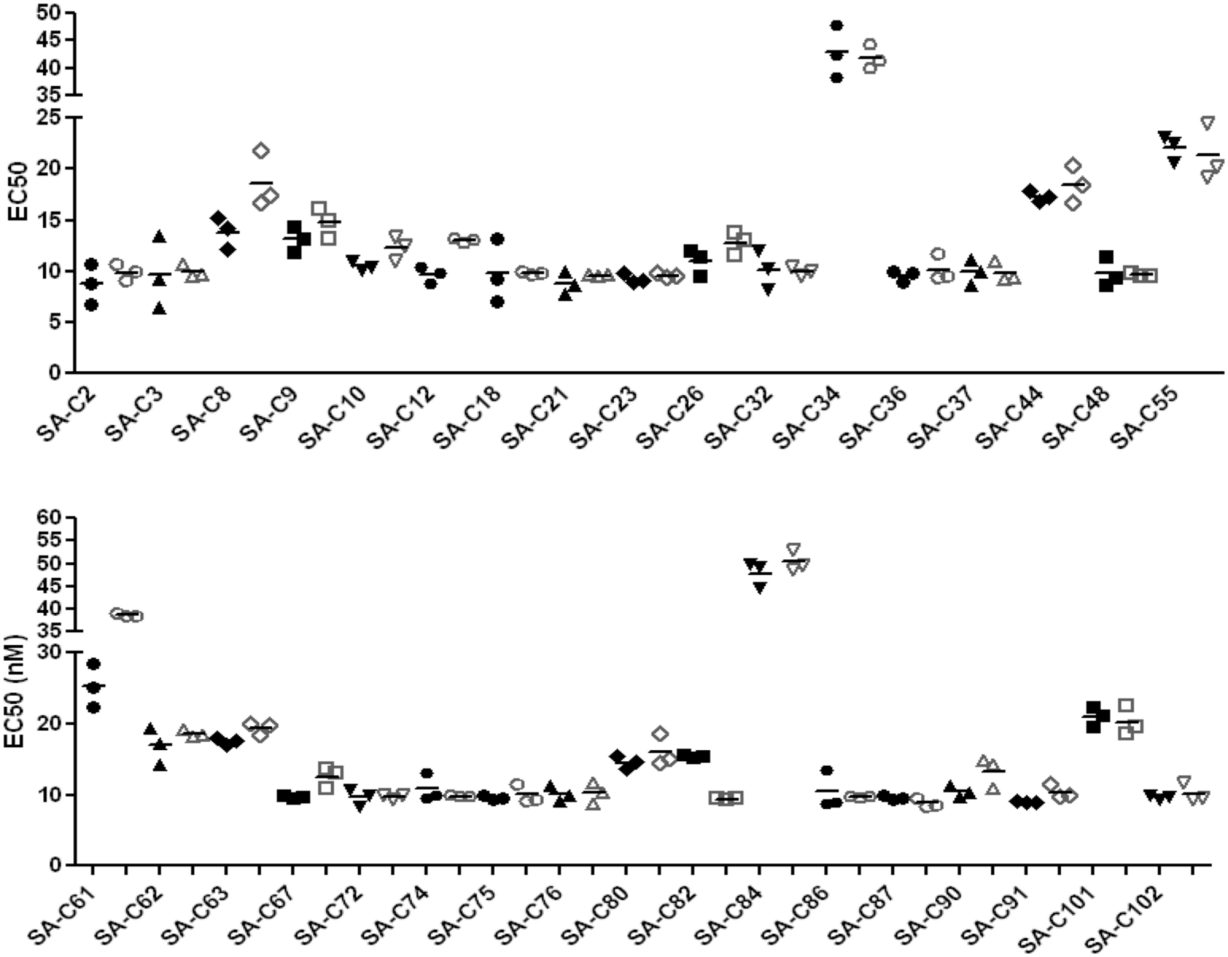
Binding of purified gp120 proteins to polyclonal sera from 34 HIV+ South African volunteers (filled symbols correspond to TV1.C gp120, open symbols to 1086.C gp120; the line indicates the mean value from triplicate measurements). All sera bound with high and comparable affinities to both gp120s, with the only exception of SA-C61 which showed higher affinity for TV1.C gp120.

We also examined the binding of the TV1.C and 1086.C gp120s to polyclonal sera from 34 HIV+ South African volunteers. Both gp120s bound to all but one of these sera with comparable EC50s (Fig 4); for SA-C61, 1086.C gp120 bound with a significantly higher EC50 (p < 0.01) than TV1.C gp120.

### Evaluation of the immunogenicity of purified monomeric and dimeric TV1.C and 1086.C gp120 proteins

**Fig 5:**
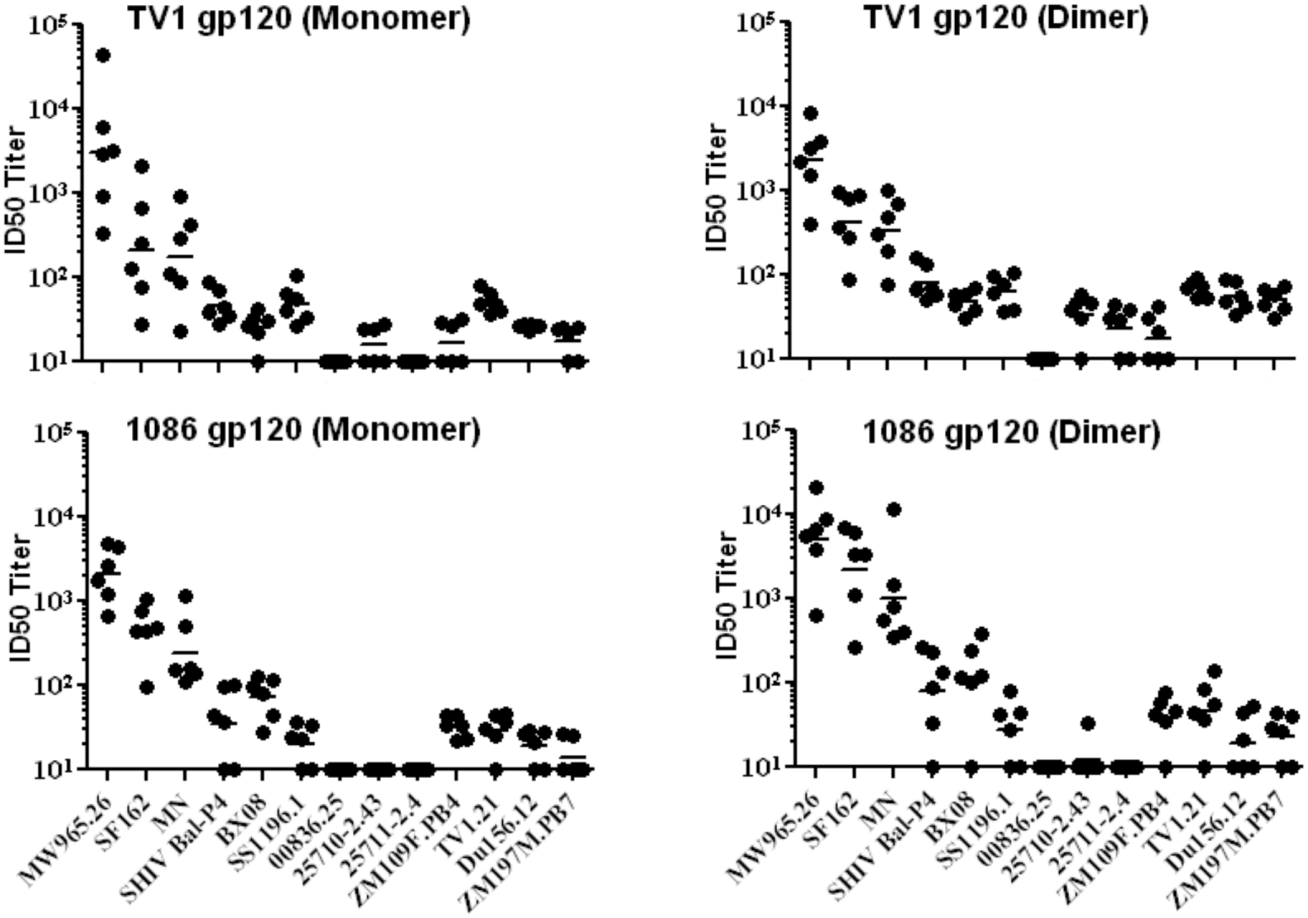
Comparative immunogenicity of TV1.C and 1086.C gp120 monomers and dimers. Rabbit immune sera collected post-3^rd^ immunization were evaluated. Serum Ab binding titers (panel A) and avidities (panel B) elicited by monomeric and dimeric fractions of TV1.C and 1086.C gp120s. Strain matched gp120 proteins were used for the binding and avidity analyses. Virus neutralization ID50 titers elicitied by monomeric (solid symbols) and dimeric (open symbols) fractions of TV1.C (panel C) and 1086.C (panel D) against a tier 1 and tier 2 HIV-1 pseudoviruses. No statistically significant differences were observed.

The immunogenicity of purified TV1.C and 1086.C gp120s formulated with the MF59 plus carbopol adjuvant [25, 40, 41] was evaluated in rabbits (Fig 5). Each of the TV1.C or 1086.C gp120 monomeric and dimeric preparations elicited similar levels of anti-gp120 binding antibodies as early as two weeks post-third immunization (Fig 5A). In addition, the avidities of the binding antibodies generated were comparable (p>0.05) (Fig 5B). When sera were evaluated for pseudovirus neutralization against a panel of predominantly Tier 1A and 1B isolates, a range of neutralization titers were measured without any significant inter-group differences in ID50 titers against any particular isolate (Fig 5C and 5D; circles and squares identify TV1.C and 1086.C, respectively; solid and open symbols correspond to monomeric or dimeric gp120, respectively). The highest neutralization titers were scored against three neutralization sensitive Tier 1A strains (MW965.26, SF162.LS, and MN). Lower ID50 titers of ≤100 were seen against seven other isolates (SHIV Bal-P4, BX08, SS196.1, ZM109F.PB4, TV1.21, Du156 and ZM197M.PB7) and the remaining three isolates (00836.25, 25710–2.43 and 25711–2.4) were not neutralized except by sera from TV1.C gp120 dimer that showed minimal neutralizing antibody activity (Fig 5C, open circles).

**Fig 6:**
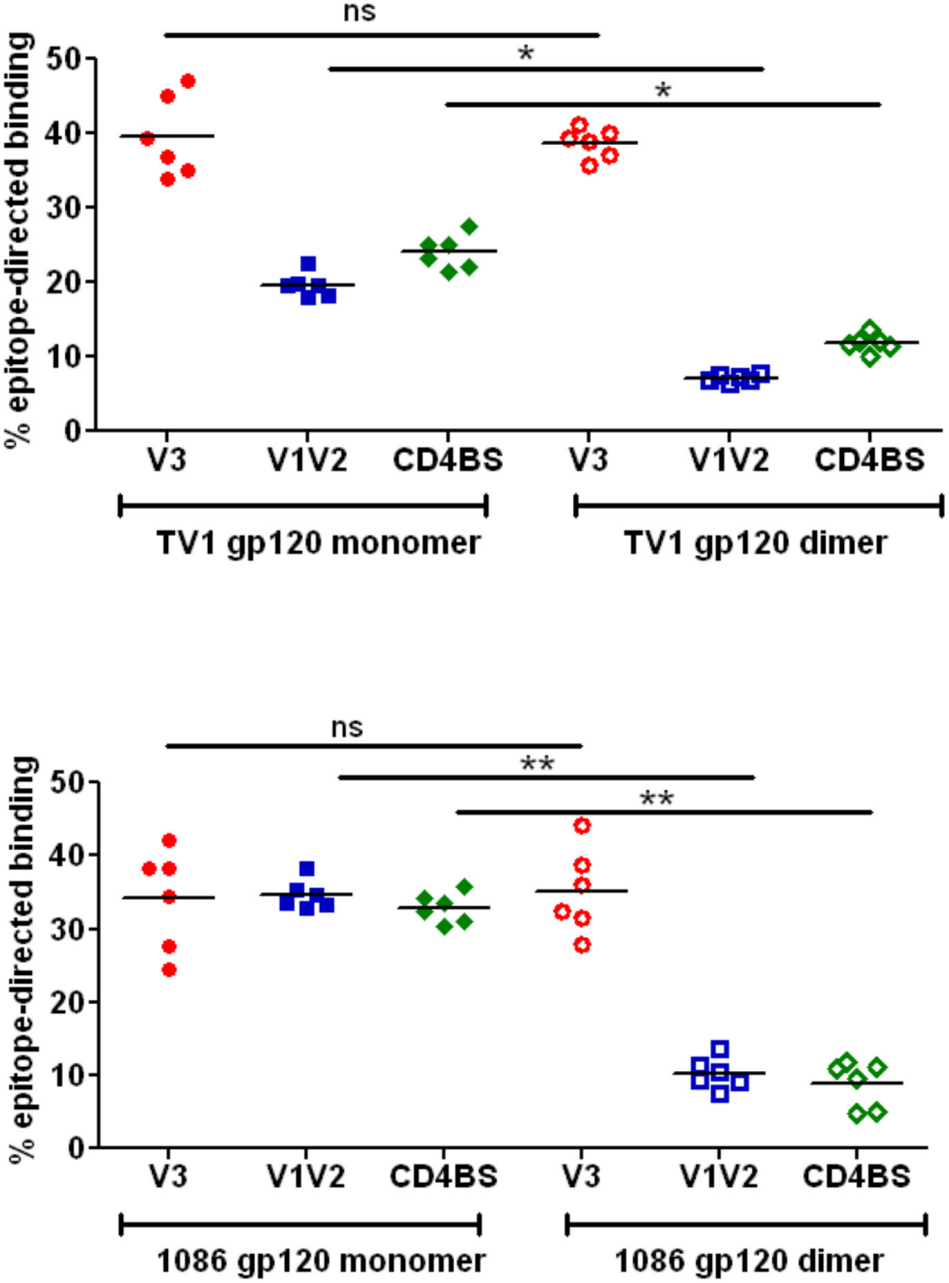
Comparison of epitope-specific binding Abs in rabbit immune sera following immunization with monomeric or dimeric TV1.C and 1086.C gp120s. V3-specific reactivity was used as a control and, as expected, showed similar binding to monomeric and dimeric gp120s. Monomeric gp120 elicited higher titers of conformational Abs directed against V1V2 and CD4BS epitopes (Ns = not significant; * = p<0.01; ** = p<0.001).

The relative levels of epitope-specific Abs in the sera on immunized rabbits were also investigated. As it might be expected from their similar binding to V3-directed mAbs, the monomeric and dimeric forms from both TV1.C and 1086.C elicited similar levels (~35–40%) of V3-reactive Abs (Fig 6A and S5A and S5D Fig). In contrast, TV1.C gp120 monomer elicited ~20% anti-V1V2 antibodies while the dimer induced only <10% V1V2-reactive Abs (p<0.01). Similarly, while ~35% of the antibodies generated by 1086.C gp120 monomer were V1V2-directed, the gp120 dimer generated significantly (p<0.001) lower levels of V1V2-directed antibodies (Fig 6B and S5B and S5E Fig). Differences in the levels of CD4BS-directed Abs elicited were also seen following immunization with monomer versus dimers; while ~25% and ≥30% of the Abs generated by TV1.C and 1086.C gp120 monomers, respectively, were directed to the CD4BS, TV1.C gp120 dimer (p<0.01) and 1086.C gp120 dimer (p<0.001) elicited significantly lower levels of CD4BS-directed antibodies (Fig 6A and 6B; S5C and S5F Fig). Thus, the antigenic differences between gp120 monomers and dimers as measured by CD4BS and V1V2 mAb binding appeared to predict the observed differences in epitope-specific Abs elicited by the two gp120 forms.

## Discussion

The results of the RV144 Phase 3 HIV vaccine trial [17] provided a rationale and key insights into potential correlates of immune protection that can be used to guide future clinical evaluations of similar prime boost regimens in regions such as southern Africa hardest hit by HIV/AIDS. To conduct these trials, it was critical that Env subunit protein vaccine candidates from HIV-1 subtype C strains, relevant to that region, be produced for the boost component of the vaccine. Therefore, two gp120 candidates from the TV1.C and 1086.C HIV-1 strains were selected and shown to have suitable properties to allow for their manufacturing at the scale required for Phase 1 and Phase 2b proof-of-concept HIV vaccine trials.

SPR and MS analyses revealed both complementarity and similarities in the antigenicity and glycan profiles of the of TV1.C and 1086.C gp120s. While both gp120s bound sCD4 and two V3-specific mAbs, they showed differential binding to CD4BS mAbs (VRC01 and b12), a mannose-dependent mAb (2G12), V1V2-directed mAbs (CH58, CH59) and a V2V3 mAb (PG09). Importantly, the presence of specific PNGs and their occupancy differed between the molecules; notably, 1086.C gp120 lacks the N160 and N332 sites recognized by broadly neutralizing Abs (bNAbs) directed to quaternary V1V2 and oligomannose patch epitopes; it should be noted that in this case, although non-occupancy of PNGs may be a direct cause of abolished mAbs binding, lack of quaternary structure can be an equally likely explanation.[42]. However, most of the N-linked as well as the single O-glycosylation sites were only partially occupied in both TV1.C and 1086.C, and 30% of the PNGs in both gp120s were decorated with primarily or exclusively high mannose glycans. Comparison of the glycosylation pattern of CHO-derived 1086.C gp120 purified here, and the previously reported, 293HEK expressed gp140, revealed that overall, the same sites were modified with similar typology of glycan (high mannose or processed) suggesting both have similar conformations proximal to these glycosylation sites.

The glycosylation profiles of these particular gp120’s show a larger number of glycosylation sites occupied exclusively with high mannose glycans compared to many other gp120’s and gp140’s that have been analyzed previously. For example, the trimeric gp140, C.ZA97012 contained only two glycosylation sites that were exclusively occupied by high mannose glycans (37). Similarly, the sequence variant gp140 JRFL contained no sites that contained exclusively high mannose glycans (35). The gp140, B.700010040.C9, which is also a transmitted/founder sequence variant had just six sites occupied exclusively with high mannose glycans (27), compared to TV1.C’s nine sites. The proportion of high mannose glycans for a given gp120 or gp140 is a variable that appears to be sequence dependent. Since it is well known that virion-derived Env has a higher proportion of high mannose glycans, the immunogens studied here, with enhanced high mannose glycans, may perform more favorably than those gp120’s and gp140’s that contain mostly processed glycoforms.

More important than measuring the absolute quantity of high mannose glycans in any given immunogen is determining whether or not the high mannose glycans are installed at the correct sites. Recently, a glycopeptide-level analysis of a trimeric-membrane anchored Env (JRFL) was conducted, and it was shown that the increase in high mannose glycans for native trimeric Env only occurs at a few key sites, while other sites retain their processed glycoforms [43]. The key sites with high mannose glycans in the membrane-anchored trimer, but not the monomer, were: N156, N262, N334, and N386-N448 [43]. By comparison, 1086C matches this profile at N262, N334, N386, N392, and N448. TV1 matches this profile at N332, N386, N392, N399, and N407. Only the N156 site was not represented with a large proportion of high mannose glycans in at least one of the two immunogens studied here. Therefore, with the exception of high mannose glycans at N156, these two immunogens together provide glycan epitopes that are similar to what one would see on a native, membrane-anchored Env trimer.

In addition, the MS analysis revealed some heterogeneous disulfide bonding, particularly in V1V2-C1, a common feature among recombinant HIV Env antigens (36–39). The dimeric gp120 fraction of TV1.C contained more extensive heterogeneity in its disulfide bonds. Homodimeric peptides, which can result only from intermolecular disulfide bonds, were observed in both C1 and the V1V2 region, suggesting that dimerization occurred in a stochastic manner involving multiple sites.

The purified TV1.C and 1086.C gp120 proteins bound well to polyclonal anti-sera from HIV-infected subjects in South African and to well-characterized human mAbs directed to the HIV Env CD4-binding site (CD4BS) and V1V2 domains. Dimeric forms did not bind well to these latter mAbs. When fractionated gp120 monomeric and dimeric forms were used to immunize rabbits using the MF59 adjuvant, they elicited high titer binding and neutralizing Abs against diverse HIV strains with titers highest against Tier 1 pseudoviruses. Of note, the monomeric gp120 preparations induced Abs that recognized CD4BS and V1V2 epitopes while the dimeric forms did not appear to efficiently elicit these specificities.

Additional studies utilizing bivalent preparations of the TV1.C and 1086.C gp120s with the MF59 adjuvant as boosts were performed in rhesus macaques comparing priming immunizations with recombinant NYVAC and ALVAC vaccine vectors [44]. Following the bivalent subtype C gp120/MF59 boost, high titer Tier 1 neutralizing responses and binding Abs were observed, the latter against both consensus subtype C gp140 and the vaccine strain gp120 antigens and also against a MuLV gp70-scaffolded V1V2 similar to that used to identify V1V2 as a potential correlate of risk in the case-controlled analysis of RV144 [19]. These findings provided further evidence that the TV1.C and 1086.C gp120s elicited Abs directed against these potentially critical epitope specificities.

It is important to note that in a recent re-analysis of the immune correlates of protection risk using a number of V2 antigens, the 1086.C V1V2 protein correlated among the best of all antigens tested [45]. Thus, the V2 of 1086.C is very similar to the V2 of the AE.A244 V2 that induced the putative protective antibodies in the RV144 trial.

In summary, these results provide support for inclusion of the TV1.C and 1086.C gp120 proteins in future clinical trials. While recent advances in the design of native trimeric HIV Env antigens [22, 23, 46, 47] may provide a pathway to the elicitation of broadly neutralizing Abs in future vaccines, it is presently of value to test the correlates of risk hypothesis from RV144. Accordingly, the two subtype C gp120s described here were advanced to cGMP production.

## Supporting Information

S1 Fig Flowchart describing gp120 adaptation and selection of gp120 CHO-based clones. (PPT)

S2 Fig Temperature experiment showing expression and viability of 1086.C gp120 clones at lowered temperature (32°C) highlighting increased specific productivity of 1086.C gp120 at lower temperature when compared to 37°C growth conditions. (PPT)

S3 Fig Consistency studies of CHO TV1.C (A, B) and 1086.C (C, D) gp120 proteins in 2 L batch re-feed bioreactors. Cell growth curves (expressed as viable cell count, 10^6^ cells/ml, left axis) and productivity (g/ml, based on HIV gp120 capture ELISA, right axis) of HIV-1 gp120 proteins in the absence of G418.Terminal batch cultures (B, D) were initiated from the corresponding parent cultures (A, C). Data points indicated by filled circles (*●*) and open circles (*○*) represent the amount of viable cell counts (10^6^ cells/ml) and total protein concentration (g/ml), respectively.
(PPT)

S4 Fig Schematic describing stability study designed to mimic GMP cell expansion and upstream production post APF and 0.5 L re-feed batch bioreactor adaptation. (PPT)

S5 Fig Overlay of ELISA binding curves to show epitope-specific binding Abs in rabbit immune sera following immunization with monomeric or dimeric TV1.C and 1086.C gp120s. The y-axis shows Optical Density (OD) read-out at 450nm; the x-axis shows serum (log) concentration. Rabbit # 1 (Ra-1) to Rabbit # 6 (Ra-6) were immunized with TV1.C gp120 monomer; Rabbit # 7 (Ra-7) to Rabbit # 12 (Ra-12) were immunized with TV1.C gp120 dimer; Rabbit # 13 (Ra-13) to Rabbit # 18 (Ra-18) were immunized with 1086.C gp120 monomer; Rabbit # 19 (Ra-19) to Rabbit # 24 (Ra-24) were immunized with 1086.C gp120 dimer. The darker color (filled and open symbols) show binding to respective V3- (A, D), V1V2- (B, E) and CD4BS- (C, F) gp120 mutants. The lighter color (filled and open) symbols indicate control binding. The data points were fitted to non-linear regression (sigmodial) analysis.
(PPTX)

S1 File (DOCX)

S1 Table Outline of selection criteria used to choose top two Subtype C gp120 proteins. five mandatory criteria and three recommended criteria were identified and acceptance criteria set to down-select the top two proteins (from several Subtype C gp120 candidates) for subsequent cell line development and gp120 production to support clinical trial material generation.
(PPT)

S2 Table Glycosylation of TV1.C gp120. The number of potential glycosylation sites and the number of occupied sites are compared for each potentially glycosylated peptide.
(PPT)

S3 Table Glycosylation of 1086.C gp120. The number of potential glycosylation sites and the number of occupied sites are compared for each potentially glycosylated peptide.
(PPT)

S4 Table Expected Disulfide Bond Pattern observed for TV1.C gp120 monomer. (PPT)

S5 Table Expected Disulfide Bond Pattern observed for 1086.C gp120 monomer. (PPT)

S6 Table Alternate Disulfide Bond Pattern observed for TV1.C gp120 monomer. (PPT)

S7 Table Alternate Disulfide Bond Pattern observed for 1086.C gp120 monomer. (PPT)

S8 Table Summary of protein purification results from five batch cultures of the TV1.C and 1086.C gp120s. (PPT)
