# Supplementary material for: Generation and Characterization of a Bivalent HIV-1 Subtype C gp120 Protein Boost for Proof-of-Concept HIV Vaccine Efficacy Trials in Southern Africa
Source: PLoS One. 2016 Jul 21;11(7):e0157391. doi: 10.1371/journal.pone.0157391 (PMC4956256; doi:10.1371/journal.pone.0157391)
Supplement: S1 Fig — (PPT) [file pone.0157391.s001.ppt]

## Slide 1
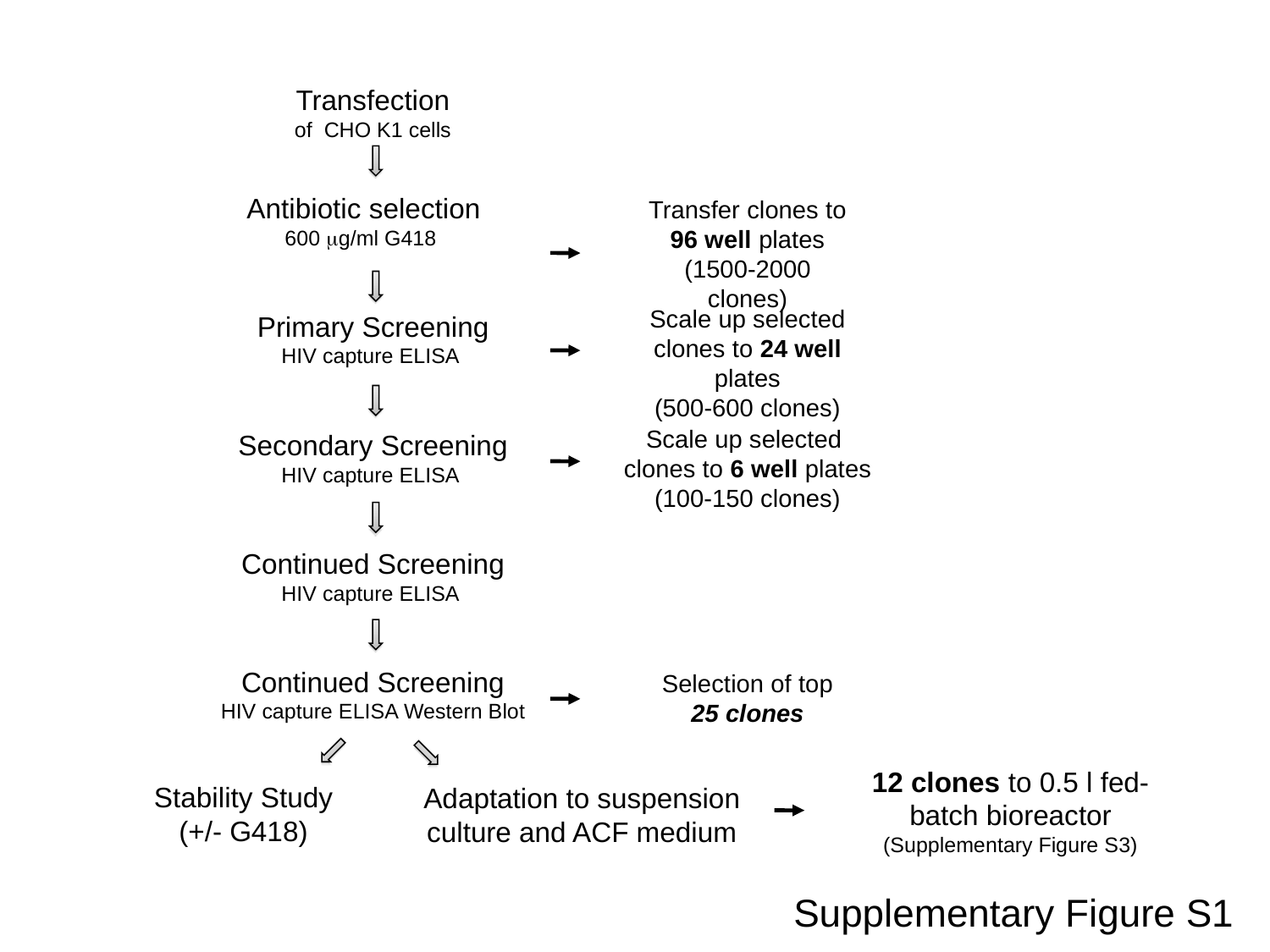

Transfection
of CHO K1 cells
Antibiotic selection
600 μg/ml G418
Transfer clones to 96 well plates
(1500-2000 clones)
Primary Screening
HIV capture ELISA
Scale up selected clones to 24 well plates
(500-600 clones)
Secondary Screening
HIV capture ELISA
Scale up selected
clones to 6 well plates
(100-150 clones)
Continued Screening
HIV capture ELISA
Continued Screening
HIV capture ELISA Western Blot
Selection of top 25 clones
12 clones to 0.5 l fed-batch bioreactor (Supplementary Figure S3)
Adaptation to suspension culture and ACF medium
Stability Study (+/- G418)
Supplementary Figure S1
