# Supplementary figures and images for: Generation and Characterization of a Bivalent HIV-1 Subtype C gp120 Protein Boost for Proof-of-Concept HIV Vaccine Efficacy Trials in Southern Africa
Source: PLoS One. 2016 Jul 21;11(7):e0157391. doi: 10.1371/journal.pone.0157391 (PMC4956256; doi:10.1371/journal.pone.0157391)

## Slide 1
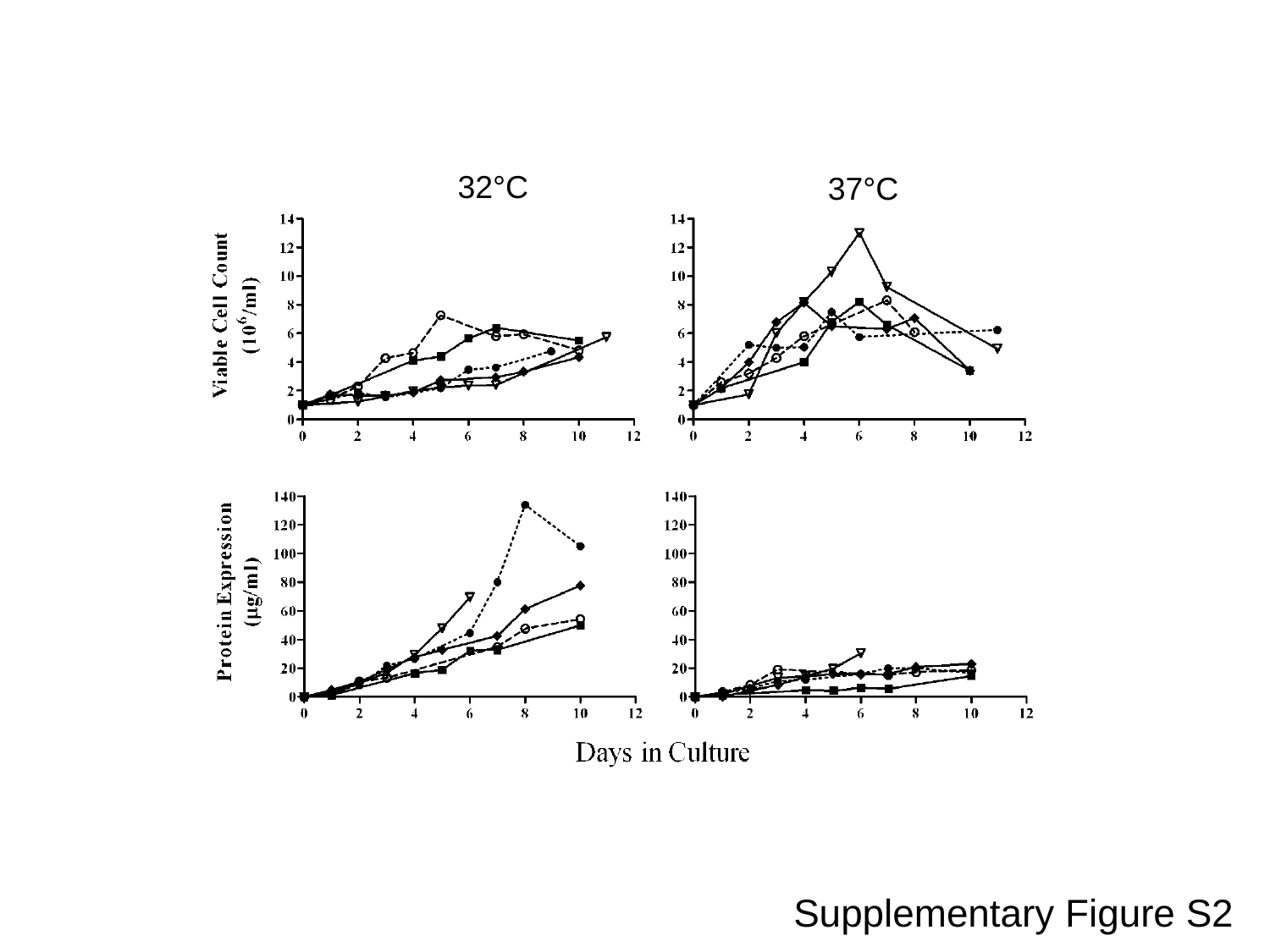

32°C
37°C
Supplementary Figure S2

Supplement: S2 Fig — (PPT) [file pone.0157391.s002.ppt]

## Slide 1
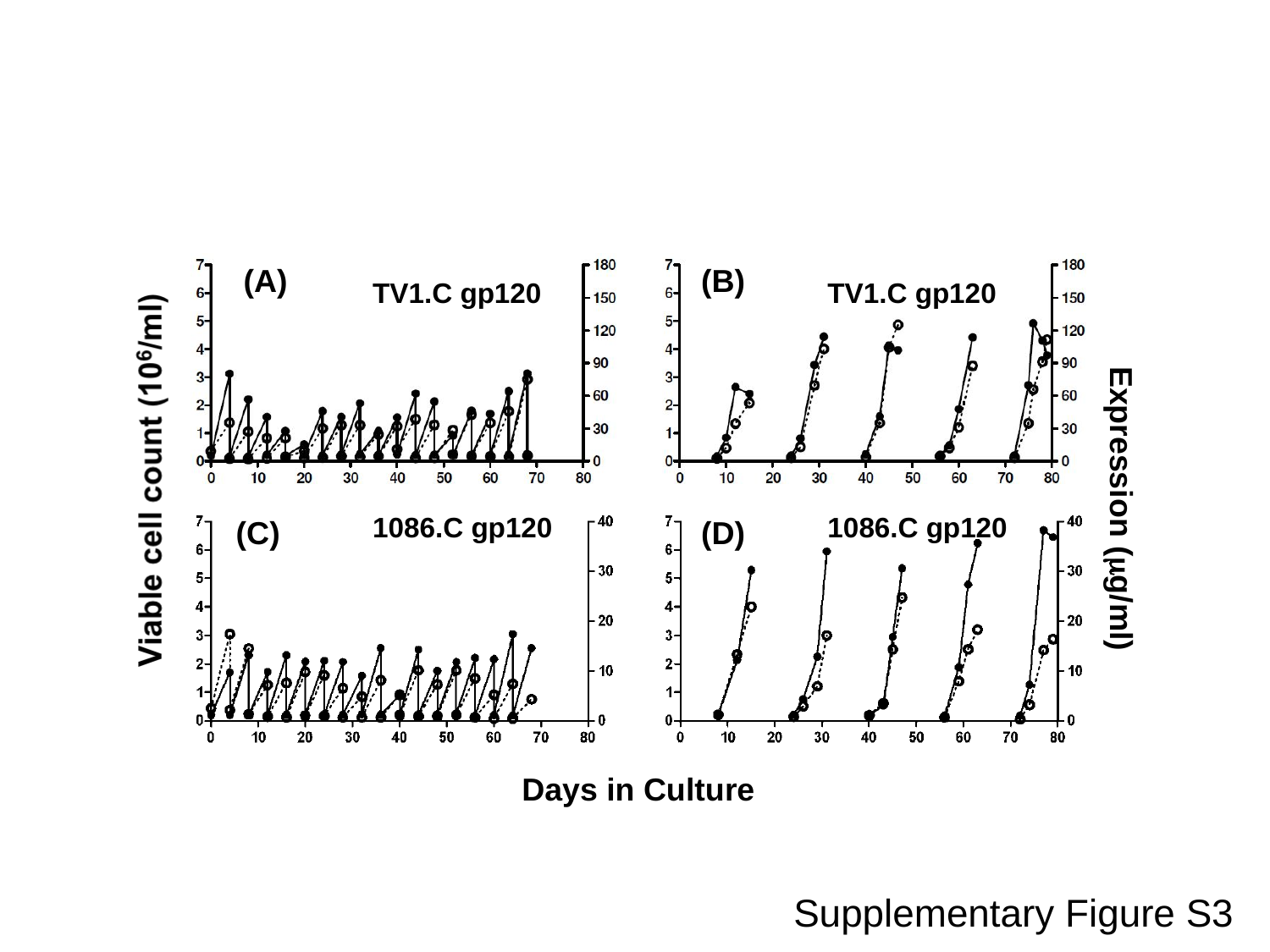

(A)
(B)
TV1.C gp120
TV1.C gp120
Expression (μg/ml)
1086.C gp120
1086.C gp120
(C)
(D)
Days in Culture
Supplementary Figure S3

Supplement: S3 Fig — Cell growth curves (expressed as viable cell count, 106 cells/ml, left axis) and productivity (g/ml, based on HIV gp120 capture ELISA, right axis) of HIV-1 gp120 proteins in the absence of G418.Terminal batch cultures (B, D) were initiated from the corresponding parent cultures (A, C). Data points indicated by filled circles (●) and open circles (○) represent the amount of viable cell counts (106 cells/ml) and total protein concentration (g/ml), respectively. (PPT) [file pone.0157391.s003.ppt]

## Slide 1
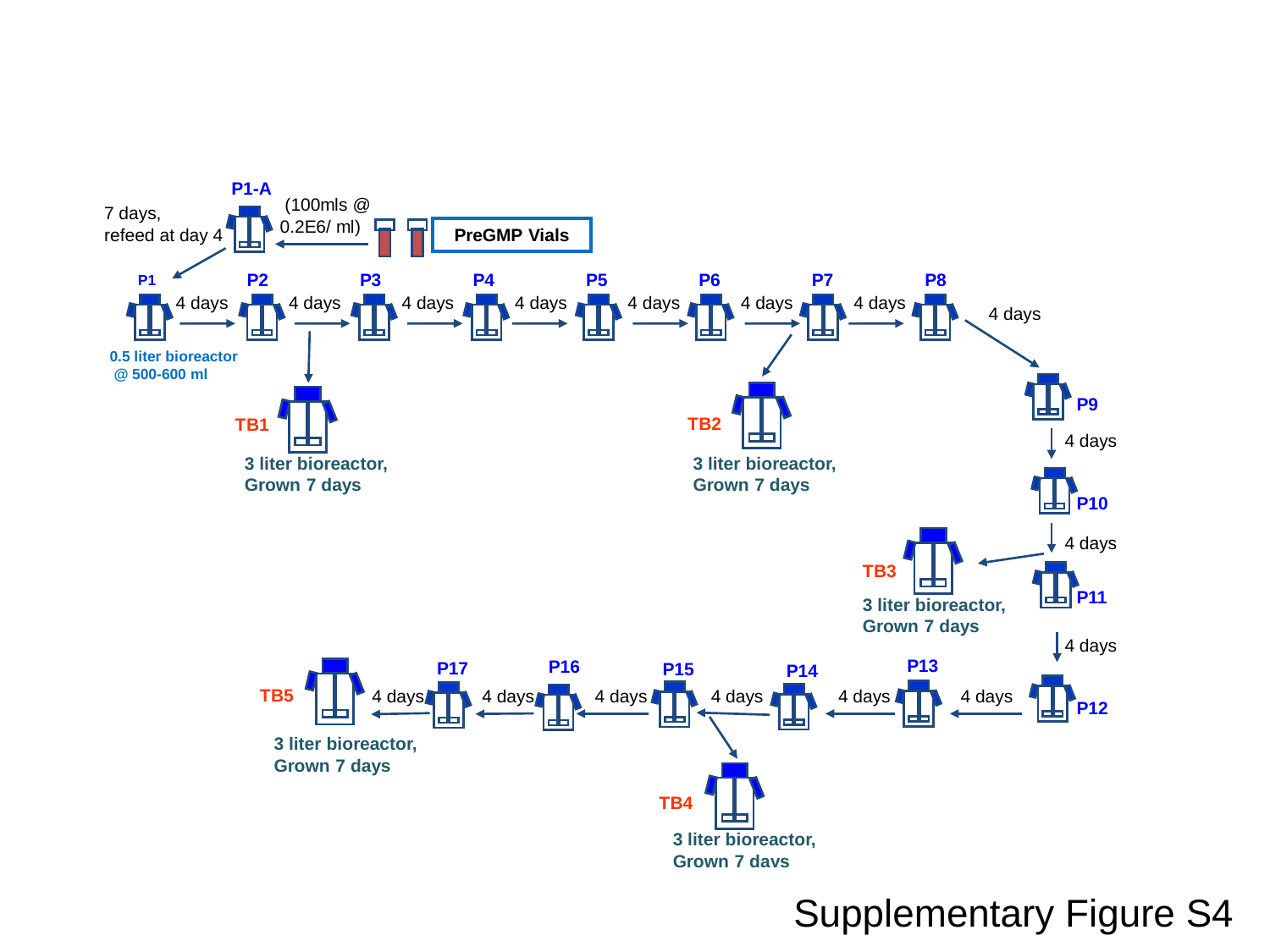

Supplementary Figure S4

Supplement: S4 Fig — (PPT) [file pone.0157391.s004.ppt]

## Slide 1
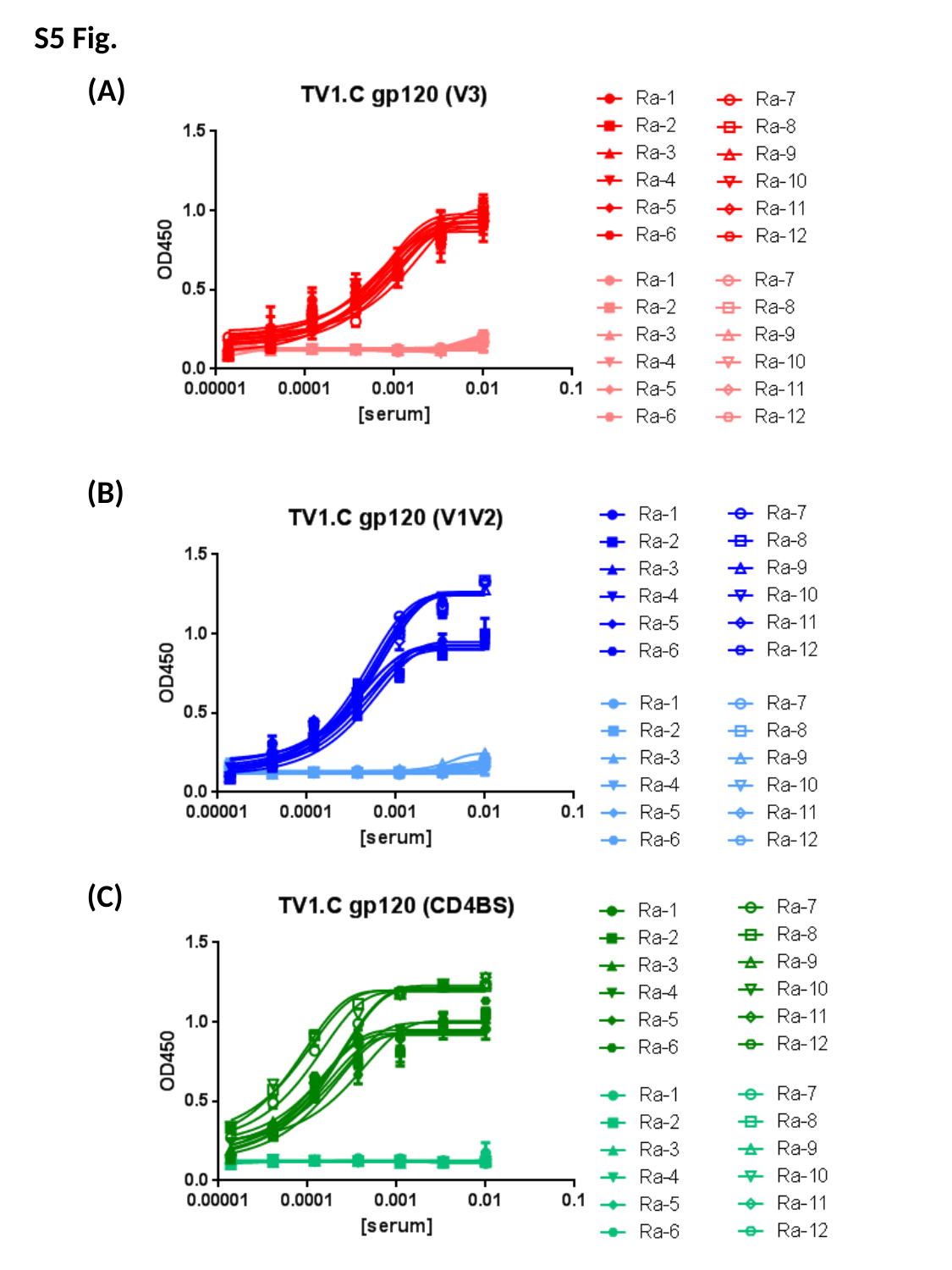

S5 Fig.
(A)
(B)
(C)

## Slide 2
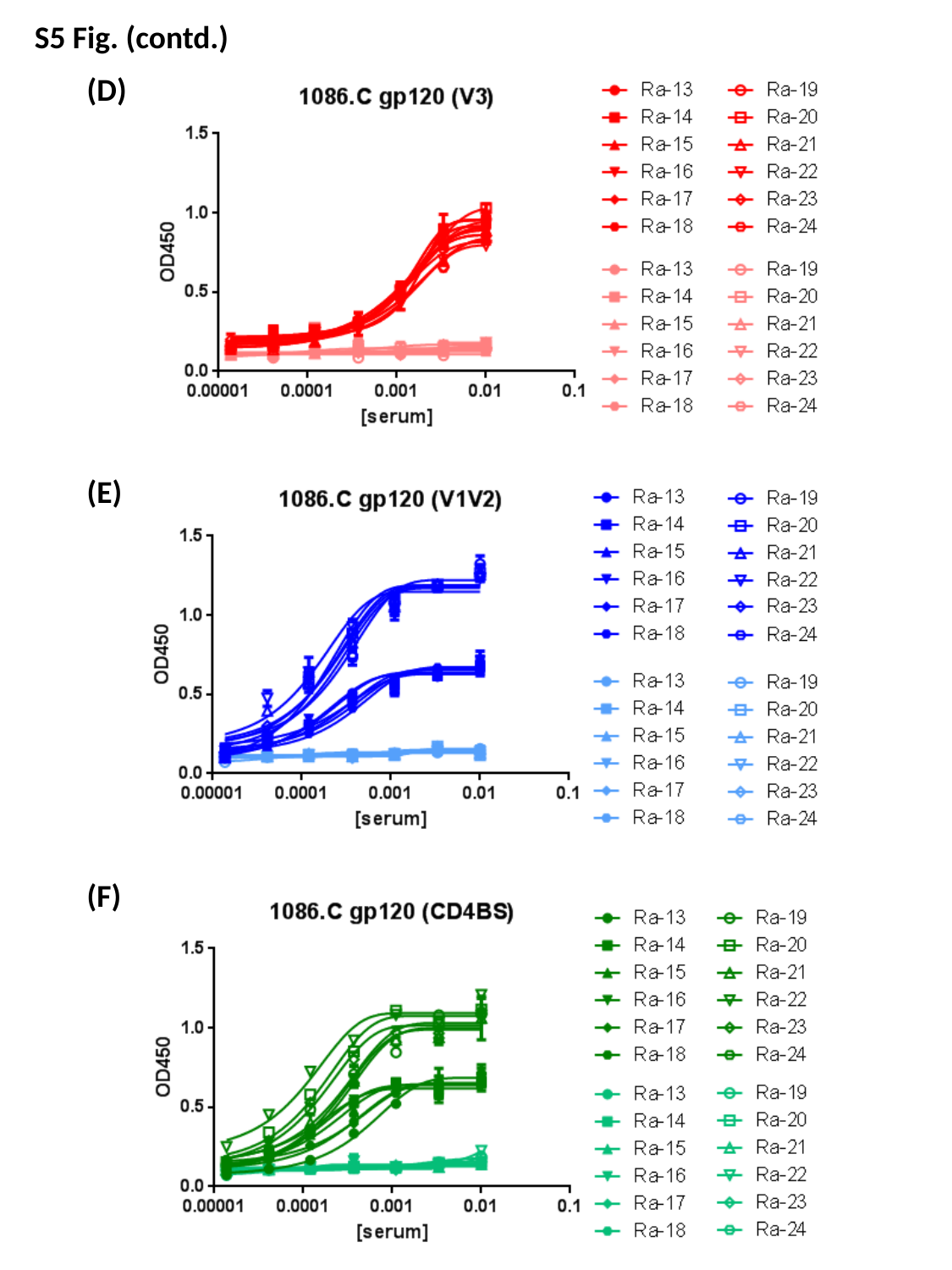

S5 Fig. (contd.)
(D)
(E)
(F)

Supplement: S5 Fig — The y-axis shows Optical Density (OD) read-out at 450nm; the x-axis shows serum (log) concentration. Rabbit # 1 (Ra-1) to Rabbit # 6 (Ra-6) were immunized with TV1.C gp120 monomer; Rabbit # 7 (Ra-7) to Rabbit # 12 (Ra-12) were immunized with TV1.C gp120 dimer; Rabbit # 13 (Ra-13) to Rabbit # 18 (Ra-18) were immunized with 1086.C gp120 monomer; Rabbit # 19 (Ra-19) to Rabbit # 24 (Ra-24) were immunized with 1086.C gp120 dimer. The darker color (filled and open symbols) show binding to respective V3- (A, D), V1V2- (B, E) and CD4BS- (C, F) gp120 mutants. The lighter color (filled and open) symbols indicate control binding. The data points were fitted to non-linear regression (sigmodial) analysis. (PPTX) [file pone.0157391.s005.pptx]

## Slide 1
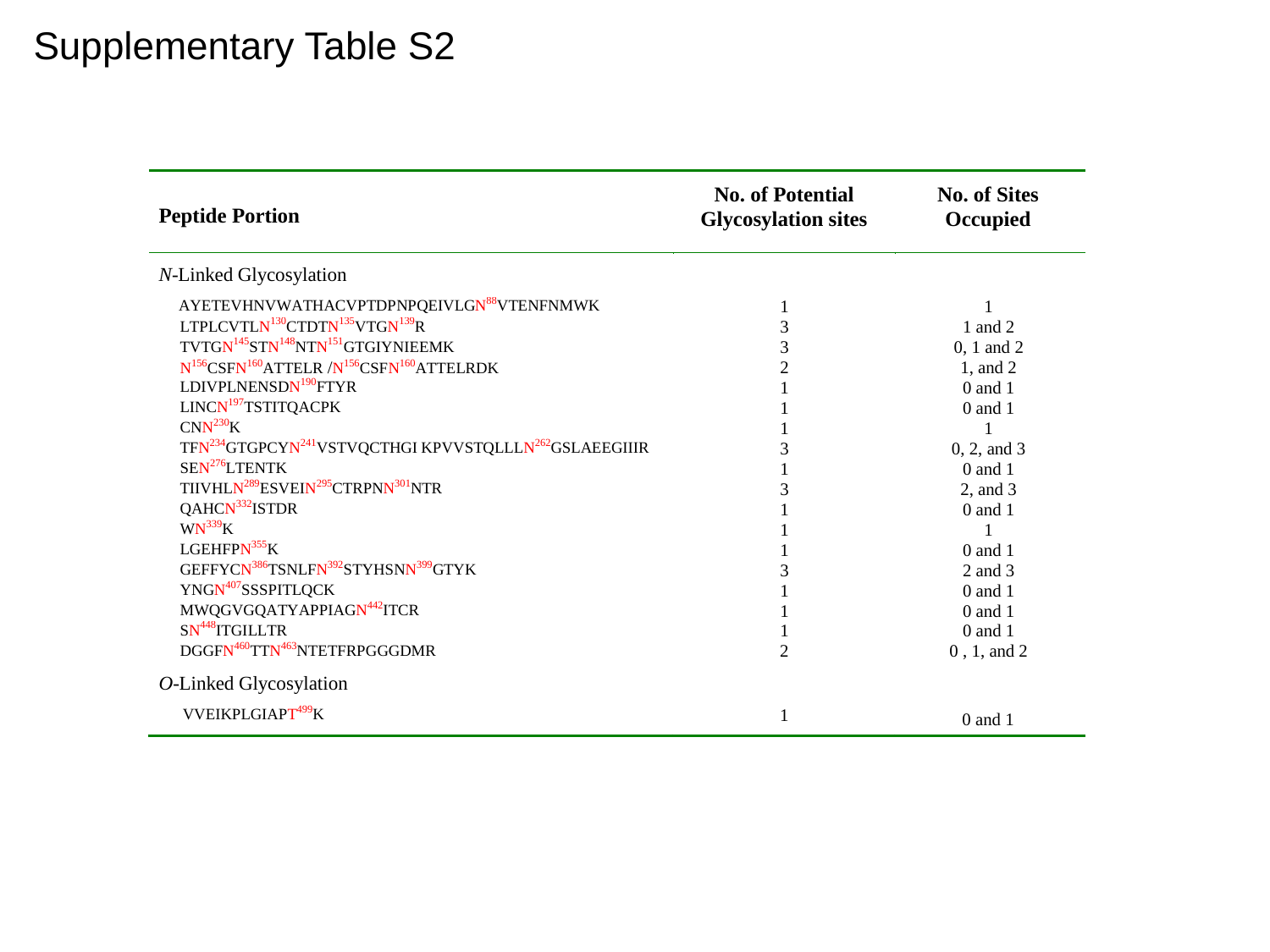

Supplementary Table S2

Supplement: S2 Table — The number of potential glycosylation sites and the number of occupied sites are compared for each potentially glycosylated peptide. (PPT) [file pone.0157391.s008.ppt]

## Slide 1
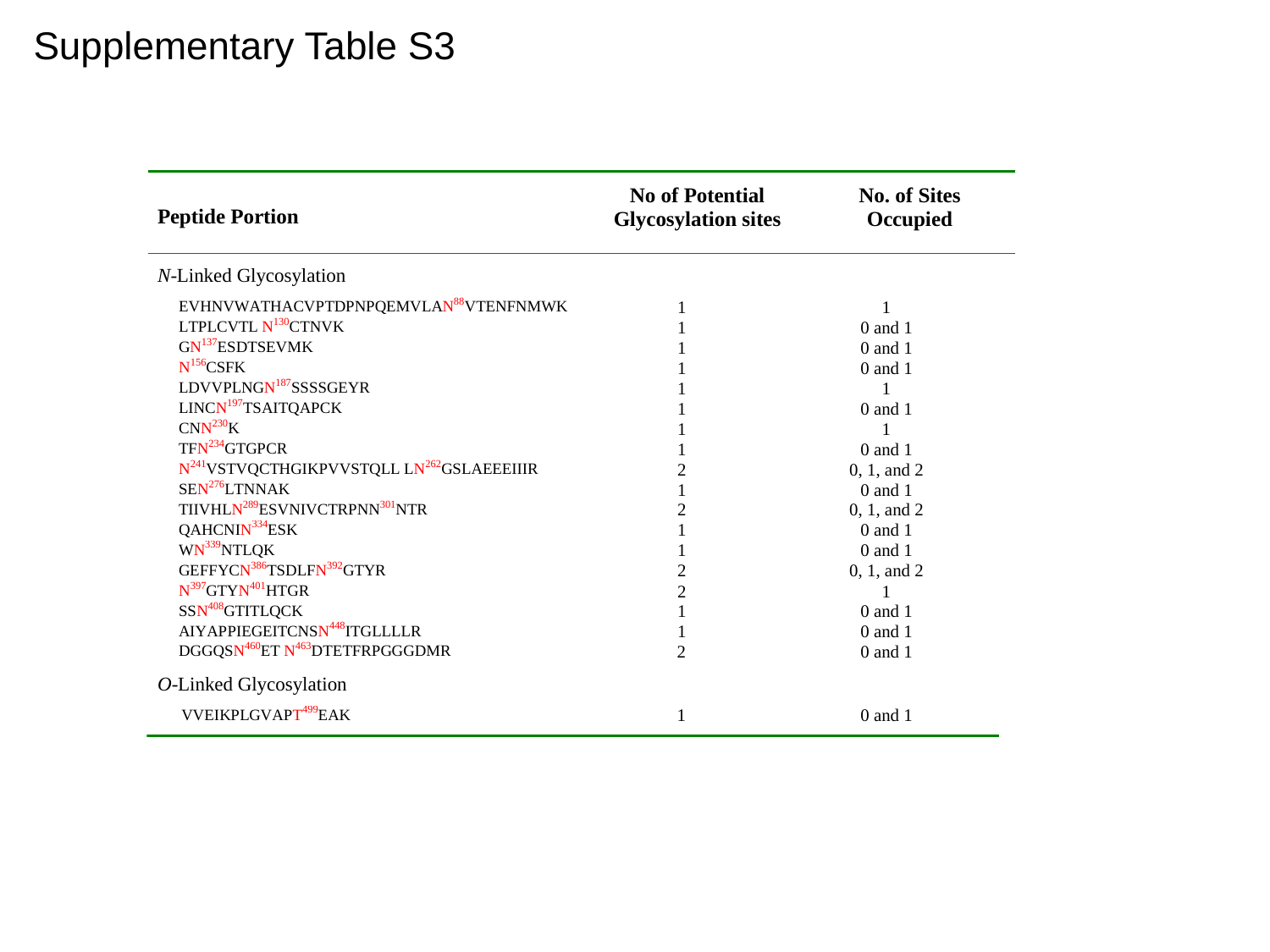

Supplementary Table S3

Supplement: S3 Table — The number of potential glycosylation sites and the number of occupied sites are compared for each potentially glycosylated peptide. (PPT) [file pone.0157391.s009.ppt]

## Slide 1
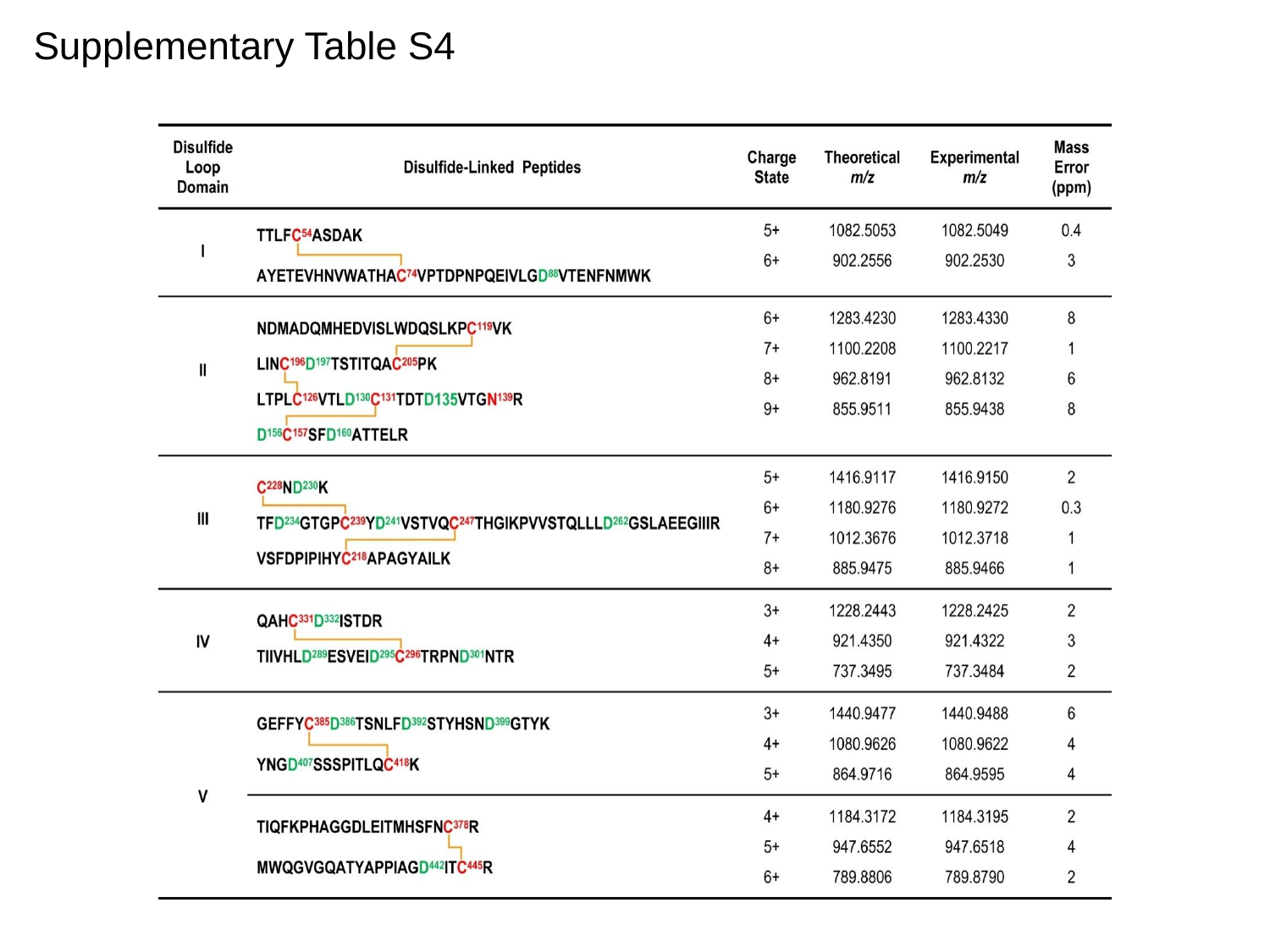

Supplementary Table S4

Supplement: S4 Table — (PPT) [file pone.0157391.s010.ppt]

## Slide 1
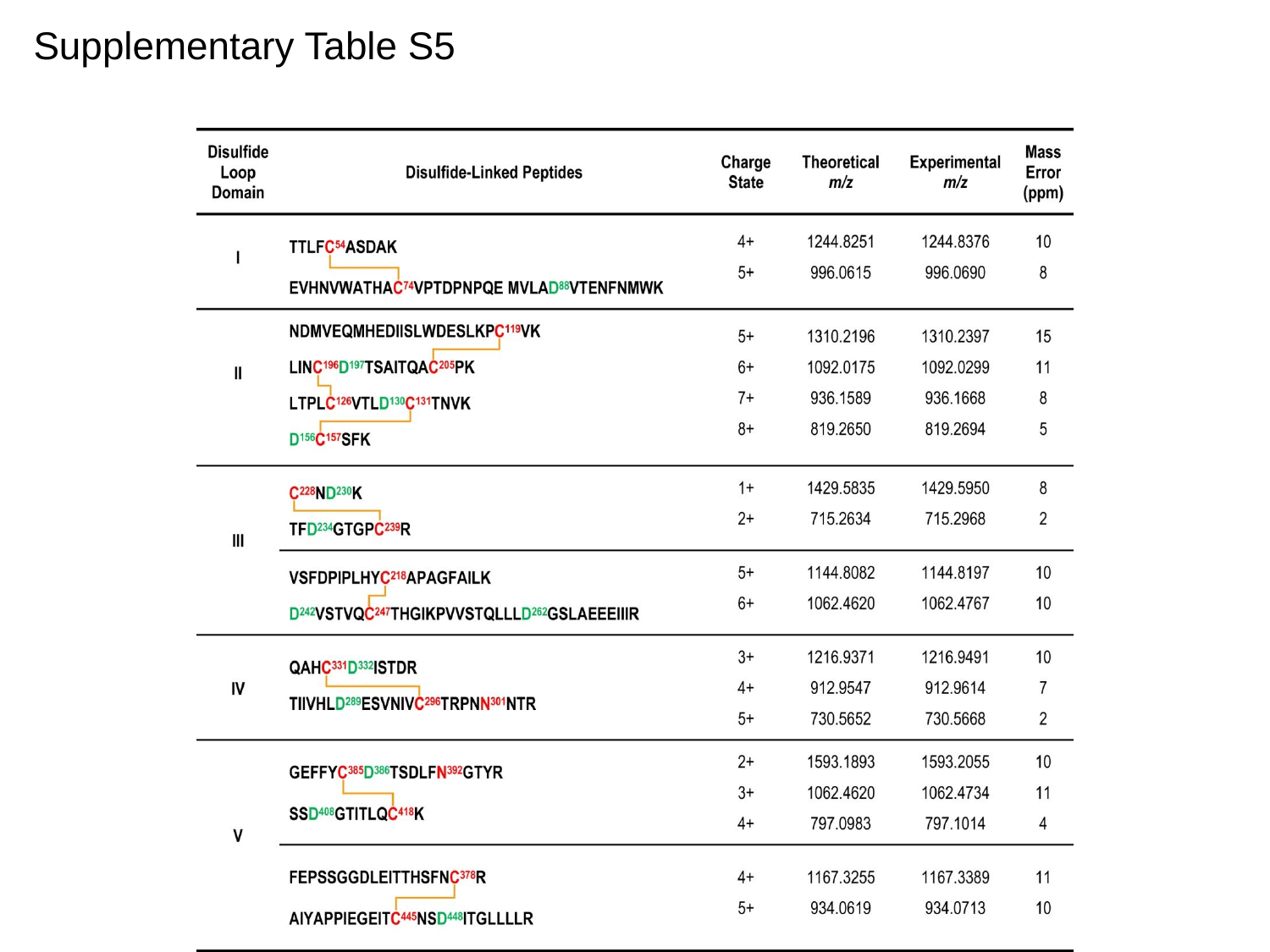

Supplementary Table S5

Supplement: S5 Table — (PPT) [file pone.0157391.s011.ppt]

## Slide 1
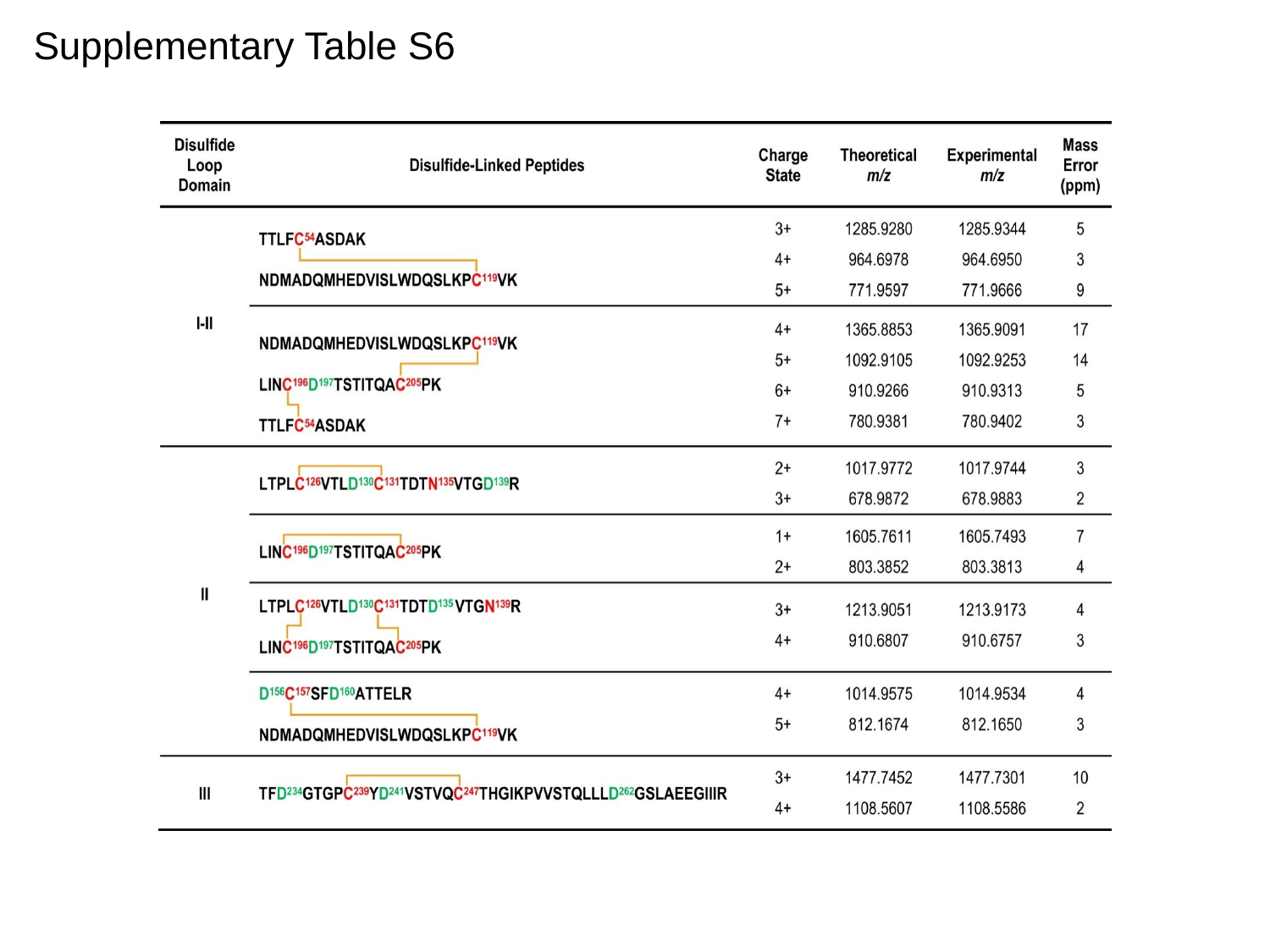

Supplementary Table S6

Supplement: S6 Table — (PPT) [file pone.0157391.s012.ppt]

## Slide 1
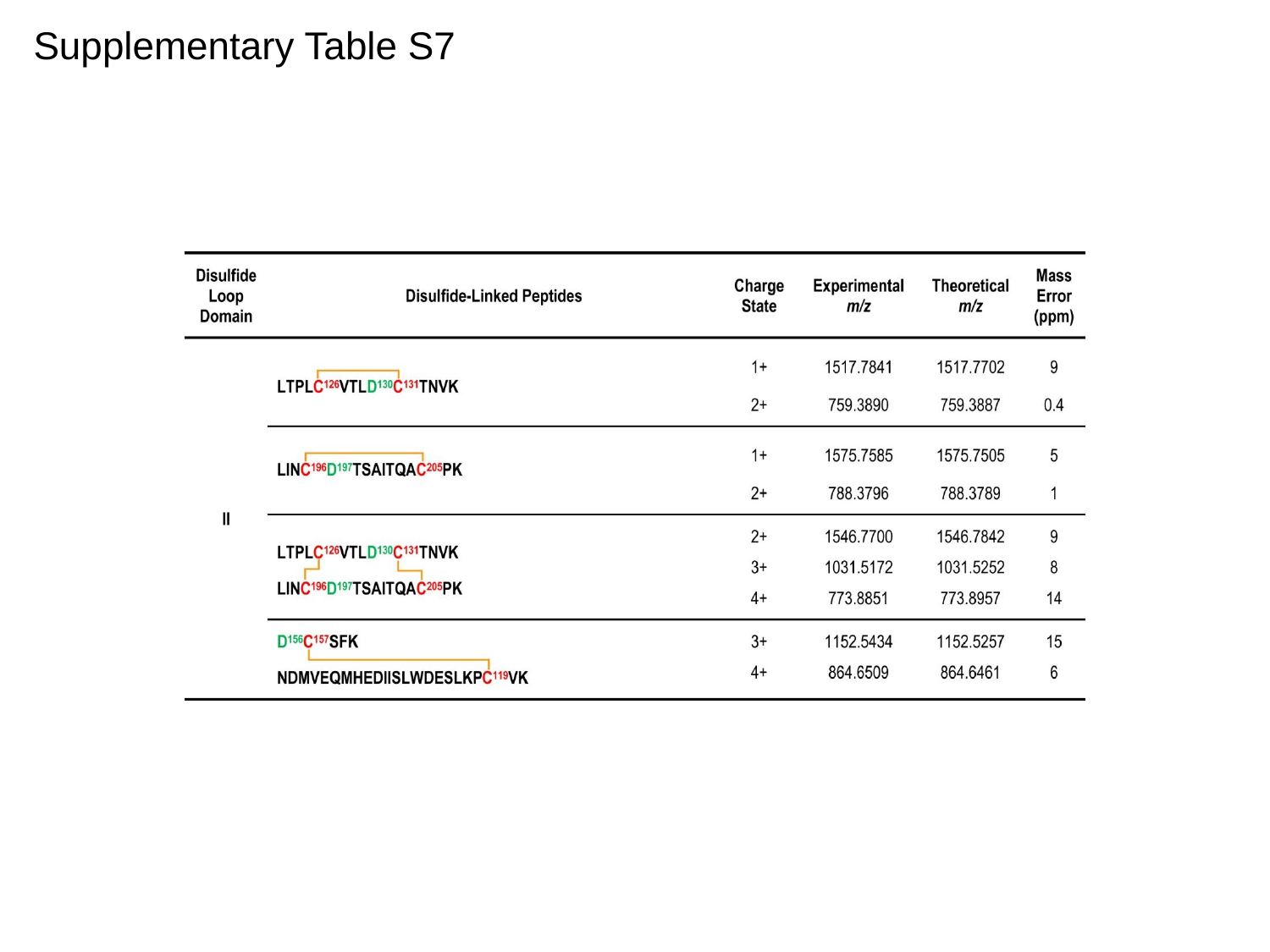

Supplementary Table S7

Supplement: S7 Table — (PPT) [file pone.0157391.s013.ppt]
