# Supplementary material for: Generation and Characterization of a Bivalent HIV-1 Subtype C gp120 Protein Boost for Proof-of-Concept HIV Vaccine Efficacy Trials in Southern Africa
Source: PLoS One. 2016 Jul 21;11(7):e0157391. doi: 10.1371/journal.pone.0157391 (PMC4956256; doi:10.1371/journal.pone.0157391)
Supplement: S1 File — (DOCX) [file pone.0157391.s006.docx]

**Generation and characterization of a bivalent HIV‑1 subtype C gp120 protein boost for future proof-of-concept HIV vaccine efficacy trials in southern Africa**

Carlo Zambonelli^1,£,#^, Antu K. Dey^1,$,#^, Susan Hilt^1,≠^, Samuel Stephenson^1^, Eden P. Go^2^, Daniel F. Clark^2^, Mark Wininger^1^, Celia Labranche^3^ , David Montefiori^3^, Hua-Xin Liao^4^, Ronald I. Swanstrom^5^, Heather Desaire^2^, Barton F. Haynes^4^, Andrea Carfi^1^* and Susan W. Barnett^1^*

*^1^GSK Vaccines, 45 Sidney Street, Cambridge, MA 02139, USA; ^2^Department of Chemistry, University of Kansas, Lawrence, KS 66047; ^3^Department of Surgery, Duke University Medical Center, Durham, North Carolina, USA; ^4^Duke Human Vaccine Institute, Duke University Medical Center, Durham, North Carolina, USA; ^5^University of North Carolina, Chapel Hill, NC, USA*

Running title: Subtype C gp120 protein vaccine

* Corresponding authors

^#^ Equal contribution

Mailing address: GSK Vaccines, 45 Sidney Street, Cambridge, MA 02139. Phone: (617) 871-8143. Fax: (617) 871-8759. E-mail: [susan.w.barnett@gsk.com](mailto:susan.w.barnett@gsk.com)

Mailing address: GSK Vaccines, 45 Sidney Street, Cambridge, MA 02139. Phone: (617) 872-8784. Fax: (617) 871-8758. Email: [andrea.x.carfi@gsk.com](mailto:andrea.x.carfi@gsk.com)

^£^ Present address: GSK Vaccines, Via Fiorentina 1, Siena 53100, Italy

^$^ Present address: International AIDS Vaccine Initiative, 125 Broad Street, 9^th^ Floor New York, NY 10004

Keywords: HIV, Subtype C, gp120, antigen selection, vaccine development, MF59

**Reagents**

CD4-Fc (sCD4) was purchased from Progenics Pharmaceuticals. Several of the monoclonal antibodies (mAbs) used here were generated in-house by cloning the sequences of V_L_ and V_H_ chains (obtained from PDB database) into an Ab-expression vector backbone containing the human C_L_ and C_H_ chains. The pDNA was then used to transfect HEK 293T cells and the expressed mAb-containing supernatants were diafiltered and concentrated 10x before purifying using Protein A affinity chromatography. MAbs CH01, CH58 and CH59 were generated as described previously and generously provided by Drs. Larry Liao and Barton Haynes [1, 2]. The 34 HIV+ sera from South African volunteers were a gift from Michael Seaman (Center for Virology and Vaccine Research, Beth Israel Deaconess Medical Center, Harvard Medical School, Boston, Massachusetts 02215, USA). The gp120 mutants (TV1.C gp120ΔV3, TV1.C gp120ΔV1V2, TV1.C gp120 D368R and 1086.C gp120ΔV3, 1086.C gp120ΔV1V2, 1086.C gp120 D368R) used in serum-mapping studies were generated as described previously [3].

**Generation of CHO stable cell lines**

The protocol for generation of stable cell lines expressing and secreting soluble gp120 is summarized in Supplementary Figure S1 and briefly described in the following paragraph.

Plasmid DNA encoding gp120 Env gene was transfected (with lipofectamine 2000CD) into CHO K1 cells and cultured in plates containing HAM’S F12 + 5% FBS + 3 mM glutamine at 37°C with 7% CO_2_. After 10 days the individual clones expressing higher levels of gp120 Env (as measured by HIV gp120 capture ELISA) were transferred to 24 well plates and the top 25 clones were transferred to 6 well plates. All cell clone expansion was carried out in selective media containing 600 µ g/ml G418 (Geneticin®). The top 25 clones were further screened for expression stability: the clones were cultured adherently in 6 well plates, with and without G418 selection and 5% FBS, for 12 weeks. Clones were assayed for expression on a weekly basis. The top 9 gp120 expressing clones were then adapted to animal component free (ACF) media (SAFC DHFR media, catalog #C8862) and suspension culture in 125 ml vented shake flasks kept at 37°C with 8% CO_2_, on an orbital shaker at 115 rpm in a humidified incubator. During adaptation, the clones were monitored for levels of gp120 expression using HIV gp120 capture ELISA. Finally, for each of the top 4 strains, 3-12 shake flask adapted clones were transferred to 0.5 L re-feed batch culture in 3 L bioreactors. Consistent stable growth (based on viable cell count, 10^6^ cells/ml) and stable gp120 expression (in µg/ml, based on HIV gp120 capture ELISA) allowed selection of the top four clones for HIV gp120 TV1.C and 1086.C.

**Clone stability and process consistency**

A 78-day stability study designed to mimic GMP cell expansion and upstream production post APF and 0.5 L re-feed batch bioreactor adaptation was carried out with HIV gp120 CHO-K1 clones (Fig. S4). The stability study comprised of two phases: 1) Continuous parent culture 600 ml re-feed batch bioreactors run sequentially for 4 days each (with and without G418 selection). Parent cultures were the inoculum for actual stability studies by terminal batch (TB) bioreactors; 2) seven day terminal batch 1,000 ml bioreactors run without G418. Cell counts, chemistries, monomer/ dimer ratio, and ELISA titers were monitored throughout the runs.

Each parent culture was split every 4 days for a total of 17 cycles (P1-17). Seven day terminal batch (TB) cultures were started at passage 3 (P3) (TB1), P7 (TB2), P10 (TB3), P15 (TB4) and P17 (TB5) and these TB cultures were later used for purification and protein characterization. All of the gp120 clones grew to high cell densities with high cell viabilities in both the parental (Supplementary Figure S3A and S3C, TV1.C and 1086.C, respectively) and terminal batch cultures (Supplementary Figure S3B and S3D, TV1.C and 1086.C, respectively); the highest gp120 producing clones also showed consistent growth stability and gp120 expression as judged by ELISA and RP-HPLC. Terminal batches also showed consistent growth and high gp120 productivity (Supplementary Figure S3B, D).

**Purification of gp120 proteins**

The development of a scalable and cost-effective process for large-scale production required devising new purification protocols for each of the two gp120s.

Culture medium containing gp120 was first spun at 4000g (20,000 rpm) in JS-24.38 Rotor in Avanti Centrifuge J30I (Beckman Coultier). Cleared starting material was concentrated 1.5x using a QuixStand Benchtop System (GE) and a hollow Fiber Cartridge (Model # UFP-100-C-4X2MA), SA: 1400cm^2^, 100 kDa cut-off (GE) and buffer exchanged 10x with a buffer containing 20 mM sodium citrate pH 5.5, 1 mM EDTA, 1 mM EGTA and stored frozen until purification. After thawing, the starting material was further spun, concentrated 5-10 fold and buffer exchanged to 20 mM sodium acetate pH 5.5 (buffer A). pH and conductivity are critical parameters and were further adjusted to ensure they were 5.5 and <3 mS/cm, respectively; the protein solution obtained was loaded on a chromatographic column containing Fractogel SO_3_ equilibrated with buffer A applying a linear flow rate equal to 22.6 cm/h. After washing the column with at least 15 column volumes, monomer gp120 was recovered either with a linear pH gradient (pH 5.5-8) or through a step elution with 20 mM sodium citrate buffer pH 5.8.

Capture of 1086.C gp120 required lowering the pH of buffer A to 5.3 and substituting sodium citrate with sodium acetate; elution was achieved with a linear gradient of buffer B (0-100%, 20 mM sodium citrate pH 5.3, 300 mM NaCl). After elution from Fractogel SO_3_, both TV1.C and 1086.C gp120 proteins were further purified on a DEAE column equilibrated with a buffer containing 20 mM Tris, pH 8, 100 mM NaCl, 1 mM EDTA, 1 mM EGTA. Prior to loading on DEAE column, the material eluted from the Fractogel SO_3_ column was adjusted to pH 7.8-8.0 by the addition of 250 mM Tris pH 8.0 and conductivity was adjusted to 11-12 mS/cm. For both TV1.C and 1086.C gp120 proteins, the DEAE column was operated in flow through mode and only contaminating proteins were retained by the ion exchange resin.

The final step in the purification protocol was concentration of purified gp120 to about 1 mg/ml and buffer exchange to formulation buffer (10 mM sodium citrate pH 6.0, 300 mM sodium chloride, 1 mM EDTA, 1 mM EGTA) using a Spectrum Labs KrosFlow Research Pump II, 30 rpm stroke, combined to a KrossFlow Digital Pressure Meter Spectrum Labs and using a MicroKros Hollow Fiber Cartridge (part # P-C1-050E-100-01N, SA 20 cm2, 50 kDa cut-off.).

Purified glycoproteins were then analyzed by SDS-PAGE (for purity) and immunoblots (for identity) where primary antibody was anti-gp140 polyclonal rabbit sera. Endotoxin levels in glycoproteins, primarily used for rabbit/guinea-pig immunogenicity studies, were measured using Endosafe® cartridges and an Endosafe®-PTSTM spectrophotometer (Charles River Laboratories International, Inc., Wilmington, MA).

**Analysis of disulfide bond using LC/ESI-FTICR Mass Spectrometry**

In brief, samples containing 75 μg of gp120 protein were alkylated with 10-fold molar excess of 4-vinylpyridine in the dark for an hour at room temperature to cap free cysteines. Alkylated samples were then deglycosylated with 1 μL PNGase F solution (500,000 units/mL) at 37°C for a week. Fully deglycosylated alkylated samples were digested with trypsin (protein to enzyme ratio of 30) overnight. To ensure reproducibility, deglycosylation and subsequent trypsin digestion were performed at least two times. Following tryptic digestion, samples were analyzed using a hybrid linear ion trap Fourier Transform-Ion Cyclotron Resonance (LTQ-FTICR, ThermoScientific, San Jose CA) mass spectrometer coupled with a Waters NanoACQUITY UltraPerformance Liquid Chromatography (UPLC) system (Waters, Milford MA) for high resolution LC/MS/MS analysis and an LTQ Velos mass spectrometer (ThermoScientific, San Jose CA) equipped with electron transfer dissociation (ETD) for low resolution LC/MS/MS analysis. Chromatographic separation for both low and high resolution LC/MS/MS analyses was performed using mobile phases consisted of solvent A: 99.9% deionized H_2_O + 0.1% formic acid and solvent B: 99.9 % CH_3_CN + 0.1% formic acid and a C18 PepMap 300^TM^ column (150mm×300 μm i.d. 5 μM, 300Å, ThermoScientific Dionex Sunnyvale, CA). Approximately 5 μl of sample was injected into the column at a flow rate of 5 μl/min using the following gradient: a linear increase to 40% B in 50 minutes then to 90% B in 10 min. The column was held at 90% B for 10 min before re-equilibration to starting conditions. Data were collected in a data dependent acquisition mode in which the five most intense ions in a high resolution survey scan in the FTICR cell were sequentially and dynamically selected for subsequent collision-induced dissociation (CID) in the LTQ linear ion trap. The LTQ Velos mass spectrometer was set up to perform experiments by alternating CID and ETD acquisition. Data dependent acquisition (DDA) was set up to acquire 10 scan events: for every one full MS scan in the mass range, 300-2000 *m/z*, each selected *m/z* in the MS scan were subjected to three MS/MS events- (a) CID, (b) ETD, and (c) CID of the charge reduced precursor in the previous ETD event. The mass spectrometric parameters used for the experiment were: spray voltage 3.0 kV, S-lens value between 45-55%, capillary temperature of 250°C, normalized collision energy of 35% for CID, and the ion-ion reaction for ETD between the precursor ion and the radical anion, fluoranthane, was set at AGC target value of 2x10^5^ and 100 msec ion-ion reaction time. To improve ETD efficiency, supplemental activation was turned on.

The LC-MS data confirmed the canonical disulfide bond between the conserved 18 cysteines in both TV1.C gp120 monomer (Supplementary Table S4) and 1086.C gp120 monomer (Supplementary Table S5) proteins. In addition to these species, several non-canonical disulfide bonded peptides were also detected; these are commonly observed in recombinantly expressed gp120 and gp140 [4-7] (See Supplementary Tables S6 and S7). Analysis of the TV1.C gp120 dimeric population showed the presence of the 9 canonical disulfide bonds seen in the gp120 monomers (both TV1.C and 1086.C, Supplementary Tables S4 and S5), plus virtually all of the noncanoncial disulfide bonds that were observed for the TV1 monomer (Supplementary Table S6) as well as several additional scrambled inter-molecular disulfide bonds, particularly in the C1-V1V2-C2 region (Figure 3). These results confirmed that gp120 dimer formation occurs via aberrant inter-molecular disulfide bonds and that removing the dimer removes a number of the aberrant structures.

**HIV-1 neutralization assays**

Briefly, a total of 200 TCID50 pseudoviruses/well were added to diluted serum samples and incubated at 37°C for 1 h. Following incubation, 10,000 cells/well in DEAE-dextran containing medium were added and incubated for 48 h at 37°C. The final concentration of DEAE-dextran was 10 µg/ml. After a 48 h incubation, 100 µl of cells was transferred to a 96-well black solid plate (Costar) for measurements of luminescence using Bright Glo substrate solution as described by the supplier (Promega). Neutralization titers are the dilution at which relative luminescence units (RLU) were reduced by 50% compared to virus control wells after subtraction of background RLU. HIV-1 Env pseudoviruses were prepared by co-transfection of 293T cells with expression plasmids containing full-length molecularly cloned gp160 env genes from a panel of HIV-1 isolates combined with an env-deficient HIV-1 backbone vector (pSG3Δenv) using FuGENE-6 HD (Roche Applied Sciences, Indianapolis, IN), as previously reported [8]. After 48 h, the cell culture supernatants containing the pseudoviruses were filtered through 0.45 µm filters and stored at -80°C until use.

**Consistency of TV1.C and 1086.C gp120 expression and purification from top clones**

To evaluate consistency in yield, purity and monomer content of the chosen TV1.C gp120 cell line, gp120 was purified from TB1, TB5 and 3 identical re-fed batch bioreactors using cells from day 78 cultures. The gp120s were purified applying the protocol described in the previous section. Overall, protein expression, purification yield and monomer content were shown to be reproducible in the 3 lots tested (Supplementary Table S8). Purification of triplicate (runs 1-3) batch cultures at day 78 and terminal batch cultures (TB3 and TB5) (total of five samples) also was carried out for the top 1086.C gp120 cell line. Protein yield and monomer content were consistent except in one instance, run 1, where lower purity and higher yield were obtained, likely due to poor equilibration either of the protein sample or of the column before the final DEAE polishing step.

**Supplementary References**

1. Liao HX, Tsao CY, Alam SM, et al. Antigenicity and immunogenicity of transmitted/founder, consensus, and chronic envelope glycoproteins of human immunodeficiency virus type 1. Journal of virology **2013**; 87:4185-201.

2. Bonsignori M, Hwang KK, Chen X, et al. Analysis of a clonal lineage of HIV-1 envelope V2/V3 conformational epitope-specific broadly neutralizing antibodies and their inferred unmutated common ancestors. Journal of virology **2011**; 85:9998-10009.

3. Dey AK, Burke B, Sun Y, et al. Elicitation of neutralizing antibodies directed against CD4-induced epitope(s) using a CD4 mimetic cross-linked to a HIV-1 envelope glycoprotein. PloS one **2012**; 7:e30233.

4. Clark DF, Go EP, Desaire H. Simple approach to assign disulfide connectivity using extracted ion chromatograms of electron transfer dissociation spectra. Analytical chemistry **2013**; 85:1192-9.

5. Go EP, Hua D, Desaire H. Glycosylation and disulfide bond analysis of transiently and stably expressed clade C HIV-1 gp140 trimers in 293T cells identifies disulfide heterogeneity present in both proteins and differences in O-linked glycosylation. Journal of proteome research **2014**; 13:4012-27.

6. Go EP, Zhang Y, Menon S, Desaire H. Analysis of the disulfide bond arrangement of the HIV-1 envelope protein CON-S gp140 DeltaCFI shows variability in the V1 and V2 regions. Journal of proteome research **2011**; 10:578-91.

7. Kassa A, Dey AK, Sarkar P, et al. Stabilizing exposure of conserved epitopes by structure guided insertion of disulfide bond in HIV-1 envelope glycoprotein. PloS one **2013**; 8:e76139.

8. Montefiori DC. Measuring HIV neutralization in a luciferase reporter gene assay. . Vol. 485. Humana Press, **2009** (Vinayaka R. Prasad GVK, eds., ed. HIV Protocols: Second Edition ).
