# Supplementary material for: Generation and Characterization of a Bivalent HIV-1 Subtype C gp120 Protein Boost for Proof-of-Concept HIV Vaccine Efficacy Trials in Southern Africa
Source: PLoS One. 2016 Jul 21;11(7):e0157391. doi: 10.1371/journal.pone.0157391 (PMC4956256; doi:10.1371/journal.pone.0157391)
Supplement: S1 Table — five mandatory criteria and three recommended criteria were identified and acceptance criteria set to down-select the top two proteins (from several Subtype C gp120 candidates) for subsequent cell line development and gp120 production to support clinical trial material generation. (PPT) [file pone.0157391.s007.ppt]

## Slide 1
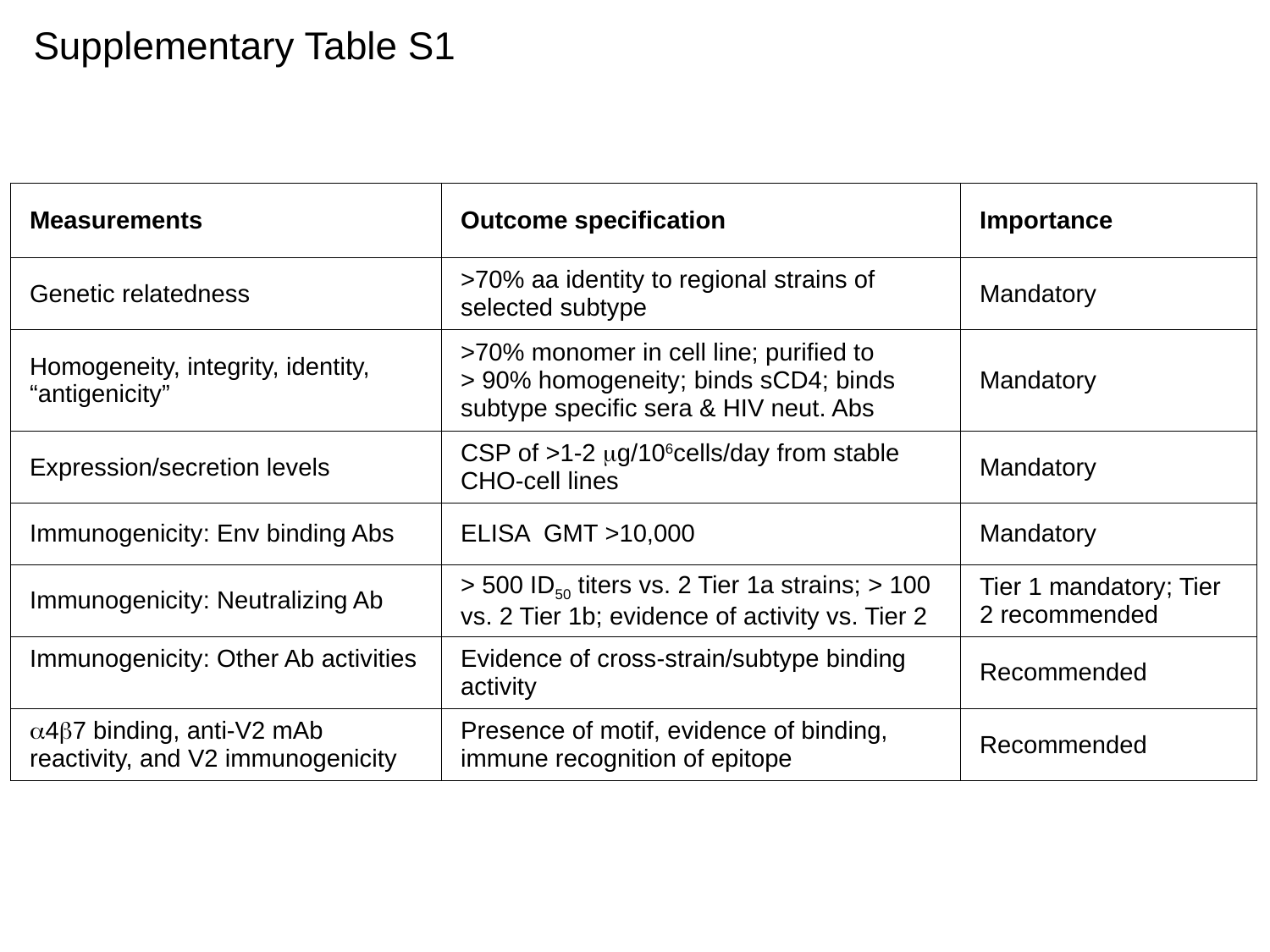

Supplementary Table S1
| Measurements | Outcome specification | Importance |
| --- | --- | --- |
| Genetic relatedness | >70% aa identity to regional strains of selected subtype | Mandatory |
| Homogeneity, integrity, identity, “antigenicity” | >70% monomer in cell line; purified to > 90% homogeneity; binds sCD4; binds subtype specific sera & HIV neut. Abs | Mandatory |
| Expression/secretion levels | CSP of >1-2 μg/106cells/day from stable CHO-cell lines | Mandatory |
| Immunogenicity: Env binding Abs | ELISA GMT >10,000 | Mandatory |
| Immunogenicity: Neutralizing Ab | > 500 ID50 titers vs. 2 Tier 1a strains; > 100 vs. 2 Tier 1b; evidence of activity vs. Tier 2 | Tier 1 mandatory; Tier 2 recommended |
| Immunogenicity: Other Ab activities | Evidence of cross-strain/subtype binding activity | Recommended |
| α4β7 binding, anti-V2 mAb reactivity, and V2 immunogenicity | Presence of motif, evidence of binding, immune recognition of epitope | Recommended |
