# Supplementary material for: Generation and Characterization of a Bivalent HIV-1 Subtype C gp120 Protein Boost for Proof-of-Concept HIV Vaccine Efficacy Trials in Southern Africa
Source: PLoS One. 2016 Jul 21;11(7):e0157391. doi: 10.1371/journal.pone.0157391 (PMC4956256; doi:10.1371/journal.pone.0157391)
Supplement: S8 Table — (PPT) [file pone.0157391.s014.ppt]

## Slide 1
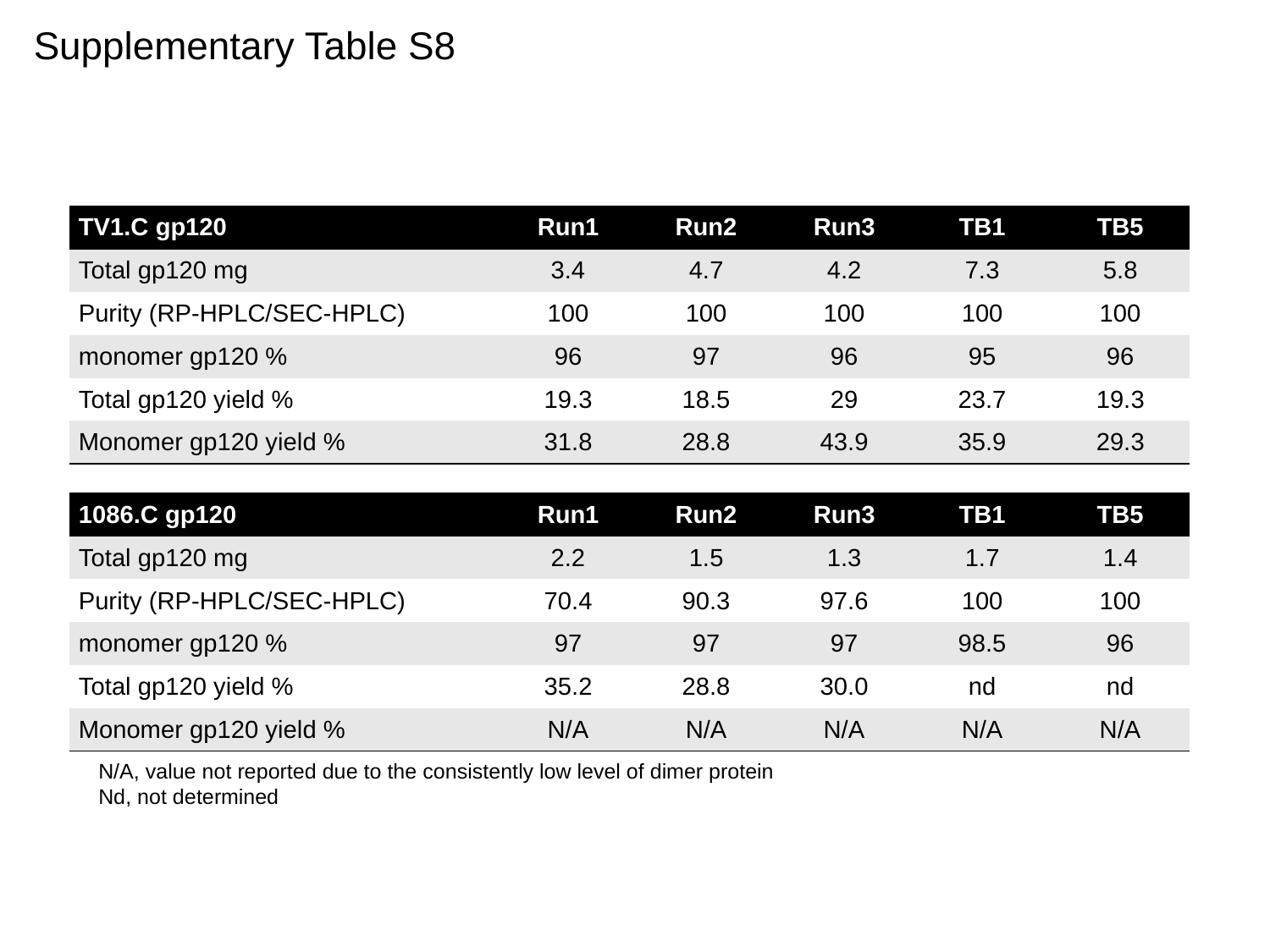

Supplementary Table S8
| TV1.C gp120 | Run1 | Run2 | Run3 | TB1 | TB5 |
| --- | --- | --- | --- | --- | --- |
| Total gp120 mg | 3.4 | 4.7 | 4.2 | 7.3 | 5.8 |
| Purity (RP-HPLC/SEC-HPLC) | 100 | 100 | 100 | 100 | 100 |
| monomer gp120 % | 96 | 97 | 96 | 95 | 96 |
| Total gp120 yield % | 19.3 | 18.5 | 29 | 23.7 | 19.3 |
| Monomer gp120 yield % | 31.8 | 28.8 | 43.9 | 35.9 | 29.3 |
| 1086.C gp120 | Run1 | Run2 | Run3 | TB1 | TB5 |
| --- | --- | --- | --- | --- | --- |
| Total gp120 mg | 2.2 | 1.5 | 1.3 | 1.7 | 1.4 |
| Purity (RP-HPLC/SEC-HPLC) | 70.4 | 90.3 | 97.6 | 100 | 100 |
| monomer gp120 % | 97 | 97 | 97 | 98.5 | 96 |
| Total gp120 yield % | 35.2 | 28.8 | 30.0 | nd | nd |
| Monomer gp120 yield % | N/A | N/A | N/A | N/A | N/A |
N/A, value not reported due to the consistently low level of dimer protein
Nd, not determined
